# Identifying primary and secondary *MLH1* epimutation carriers displaying low-level constitutional *MLH1* methylation using droplet digital PCR and genome-wide DNA methylation profiling of colorectal cancers

**DOI:** 10.1186/s13148-023-01511-y

**Published:** 2023-06-03

**Authors:** Jihoon E. Joo, Khalid Mahmood, Romy Walker, Peter Georgeson, Ida Candiloro, Mark Clendenning, Julia Como, Sharelle Joseland, Susan Preston, Lise Graversen, Mathilda Wilding, Michael Field, Michelle Lemon, Janette Wakeling, Helen Marfan, Rachel Susman, Joanne Isbister, Emma Edwards, Michelle Bowman, Judy Kirk, Emilia Ip, Lynne McKay, Yoland Antill, John L. Hopper, Alex Boussioutas, Finlay A. Macrae, Alexander Dobrovic, Mark A. Jenkins, Christophe Rosty, Ingrid M. Winship, Daniel D. Buchanan

**Affiliations:** 1 Colorectal Oncogenomics Group, Department of Clinical Pathology, Victorian Comprehensive Cancer Centre, The University of Melbourne, 305 Grattan Street, Parkville, VIC 3000 Australia; 2 Victorian Comprehensive Cancer Centre, University of Melbourne Centre for Cancer Research, Parkville, VIC Australia; 3 Melbourne Bioinformatics, The University of Melbourne, Melbourne, VIC Australia; 4 Beacon Biomarkers Lab, Department of Surgery, Austin Health, University of Melbourne, Heidelberg, VIC Australia; 5 Department of Clinical Genetics, Aarhus University Hospital, Aarhus, Denmark; 6 Department of Clinical Genetics, Royal North Shore Hospital, Sydney, NSW Australia; 7 Genetic Health Queensland, Royal Brisbane and Women’s Hospital, Herston, QLD Australia; 8 Tasman Health Care, Southport, QLD Australia; 9 Genomic Medicine and Family Cancer Clinic, Royal Melbourne Hospital, Parkville, Melbourne, VIC Australia; 10 Familial Cancer Service, Crown Princess Mary Cancer Centre, Westmead Hospital, Sydney, NSW 2145 Australia; 11 Department of Cancer Genetics, Liverpool Hospital, Liverpool, NSW Australia; 12 The Cabrini Family Cancer Clinic, Cabrini Health, Malvern, VIC Australia; 13 Centre for Epidemiology and Biostatistics, Melbourne School of Population and Global Health, The University of Melbourne, Carlton, VIC Australia; 14 Department of Gastroenterology, The Alfred Hospital, Melbourne, Parkville, VIC 3010 Australia; 15 Central Clinical School, Monash University, Melbourne, VIC 3004 Australia; 16 Colorectal Medicine and Genetics, The Royal Melbourne Hospital, Parkville, VIC Australia; 17 Department of Medicine, The University of Melbourne, Parkville, Australia; 18 Envoi Specialist Pathologists, Brisbane, Australia; 19 University of Queensland, Brisbane, Australia

**Keywords:** *MLH1* epimutation, Genome wide DNA methylation, *MLH1* methylation, MMR deficiency, Colorectal cancer, Lynch syndrome

## Abstract

**Background:**

*MLH1* epimutation is characterised by constitutional monoallelic *MLH1* promoter hypermethylation, which can cause colorectal cancer (CRC). Tumour molecular profiles of *MLH1* epimutation CRCs were used to classify germline *MLH1* promoter variants of uncertain significance and *MLH1* methylated early-onset CRCs (EOCRCs). Genome-wide DNA methylation and somatic mutational profiles of tumours from two germline *MLH1*: c.-11C > T and one *MLH1*: c.-[28A > G; 7C > T] carriers and three *MLH1* methylated EOCRCs (< 45 years) were compared with 38 reference CRCs. Methylation-sensitive droplet digital PCR (ddPCR) was used to detect mosaic *MLH1* methylation in blood, normal mucosa and buccal DNA.

**Results:**

Genome-wide methylation-based *Consensus Clustering* identified four clusters where the tumour methylation profiles of germline *MLH1*: c.-11C > T carriers and *MLH1* methylated EOCRCs clustered with the constitutional *MLH1* epimutation CRCs but not with the sporadic *MLH1* methylated CRCs. Furthermore, monoallelic *MLH1* methylation and *APC* promoter hypermethylation in tumour were observed in both *MLH1* epimutation and germline *MLH1*: c.-11C > T carriers and *MLH1* methylated EOCRCs. Mosaic constitutional *MLH1* methylation in *MLH1*: c.-11C > T carriers and 1 of 3 *MLH1* methylated EOCRCs was identified by methylation-sensitive ddPCR.

**Conclusions:**

Mosaic *MLH1* epimutation underlies the CRC aetiology in *MLH1*: c.-11C > T germline carriers and a subset of *MLH1* methylated EOCRCs. Tumour profiling and ultra-sensitive ddPCR methylation testing can be used to identify mosaic *MLH1* epimutation carriers.

**Supplementary Information:**

The online version contains supplementary material available at 10.1186/s13148-023-01511-y.

## Background

Colorectal cancer (CRC) is the third most diagnosed cancer and the second leading cause of cancer-related death, responsible for ~ 10% of all cancer incidences and cancer-related deaths worldwide [1]. DNA methylation [2], together with inherited genetic predispositions, adverse environmental risk factors and ageing [3], plays an important role in CRC aetiology. Aberrant DNA methylation changes can be detected in virtually all CRC tumours [2], but it is the transcriptional silencing of *MLH1* through promoter hypermethylation (referred to as *MLH1* methylation) that is one of the most clinically important and well-characterised epigenetic events, seen in 10–20% of all CRCs [4].

Somatically acquired biallelic *MLH1* methylation in CRC results in loss of immunohistochemical expression of the MLH1 and PMS2 DNA mismatch repair (MMR) proteins and microsatellite instability within the tumour (i.e. MMR-deficiency). Sporadic *MLH1* methylation is associated with an older age of CRC diagnosis, females and features of the serrated pathway of neoplasia [5] namely the co-existence of somatic *BRAF* p.V600E mutations and genome-wide hypermethylation of tumour suppressor genes, commonly referred to as high levels of CIMP (CpG Island Methylator Phenotype) [6]. A second sporadic subtype of MMR-deficient CRC is caused by biallelic somatic mutations in one of the DNA MMR genes (often referred to as double somatic MMR mutations) [7]. In contrast, CRCs related to Lynch syndrome (LS) result from a germline pathogenic variant in one of the DNA MMR genes and a second somatic hit causing tumour MMR-deficiency. Rarely, constitutional mismatch deficiencies (CMMRD) occur when an individual inherits two germline pathogenic variants in the same MMR gene, leading to the loss of both alleles [8]. Lynch-related MLH1 deficiency occurs in the absence of both *MLH1* methylation and features of the serrated neoplasia pathway (no *BRAF* p.V600E or CIMP-high) [9]. Therefore, tumour *MLH1* methylation testing is used as the routine diagnostics testing to differentiate sporadic *MLH1* methylated CRCs from inherited MLH1-deficient CRC caused by germline pathogenic variants (Lynch syndrome) [10, 11]. To further distinguish *MLH1* epimutation CRCs from common sporadic *MLH1* methylation CRCs, *MLH1* methylation testing of non-tumour DNA sources (e.g. blood) is recommended [12].

A rarer subtype of MMR-deficient CRC is related to constitutional hypermethylation of the *MLH1* gene promoter*,* referred to as *MLH1* epimutation. *MLH1* epimutations are characterised by monoallelic *MLH1* promoter methylation [13], resulting from either idiopathic de novo methylation (“primary epimutation”) or from a *cis-acting* genetic variant (“secondary epimutation”), which determines the transgenerational transmissibility [14]. Primary and secondary *MLH1* epimutations both present with tumour *MLH1* methylation and resultant tumour MMR deficiency. In *MLH1* epimutation carriers, soma-wide *MLH1* methylation occurs in a monoallelic manner [15], although mosaic patterns have been described [16].

The prevalence of *MLH1* epimutations is thought to be between 3 and 16% in Lynch-suspected cases with MLH1-deficient CRCs [10, 17–19]. There is currently a lack of consensus on the triaging approach to detect *MLH1* epimutation carriers, largely due to highly variable inheritance and potentially mosaic constitutional methylation patterns [10]. Testing for *MLH1* epimutation has been recommended in CRC cases diagnosed < 60 years with an *MLH1* methylated tumour and those with a history of more than one Lynch-associated tumour [10], although it is unclear how routinely these criteria are applied, primarily due to their rarity.

Adding to the complexity, cases demonstrating mosaic patterns of constitutional *MLH1* epimutation have been previously reported [16, 19]. Mosaic constitutional methylation has also been seen in other key cancer risk genes including *BRCA1* [20] and *RAD51C* [21] in levels as low as 0.01% in non-neoplastic tissue and blood DNA samples from breast and ovarian cancer cases. Though primary *MLH1* epimutations are largely thought to arise de novo, there has been a report of an early-onset colon cancer case who inherited a constitutional *MLH1* epimutation from their asymptomatic mother who had low-level (3–5%) gonosomal mosaic *MLH1* epimutation [22]. Therefore, identifying mosaic *MLH1* epimutations poses a clinical challenge for assessing not only second primary cancer risks but also cancer risks in family members. The low *MLH1* methylation levels present in mosaic cases are unlikely to be detectible by the Methylation-specific Multiplex Ligation-Dependent Probe Amplification (MS-MLPA) testing method commonly utilised in the clinical setting, highlighting the need for studies applying highly sensitive techniques such as methylation-sensitive droplet digital polymerase chain reaction (ddPCR) [23].

To date, the *MLH1*: c.-27C > A germline pathogenic variant is the only reported variant known to underlie secondary *MLH1* epimutations [14]. Several other non-coding *MLH1* promoter germline variants have been reported (e.g. c.-11C > T [19], c.-[28A > G; 7C > T] [24], c.-42C > T [25]), although their pathogenicity and effect on inducing *MLH1* methylation are less defined and, as such, remain classified as variants of uncertain clinical significance (VUS). In CRCs associated with these VUS, a constitutional reduction of *MLH1* expression was observed [19, 24, 25] but without a clear effect on *MLH1* promoter methylation. Due to their rarity, studies of these variants are scarce and validation difficult, which impedes optimal clinical management in carriers.

Differentiating *MLH1* epimutations from sporadic *MLH1* methylated CRCs has important consequences for the clinical management of patients including prevention of second primary cancers and cancer prevention in relatives [10, 26]. This study investigated the genome-wide DNA methylation and somatic mutation profiles from clinically relevant subtypes of MMR-deficient CRCs, including those defined by sporadic *MLH1* methylation or by constitutional *MLH1* epimutation. The unique DNA methylation signatures demonstrated by the sporadic *MLH1* methylated and constitutional *MLH1* epimutation tumours were investigated in CRCs from carriers of a germline VUS in the *MLH1* promoter or with tumour *MLH1* methylation in an early-onset CRC (EOCRC) to support classification. Detection of low-level *MLH1* methylation in blood and normal colonic tissue by ddPCR supported mosaic constitutional *MLH1* epimutation for these clinically challenging cases.

## Methods

### Study participants and CRC tumour samples

**Fig. 1:**
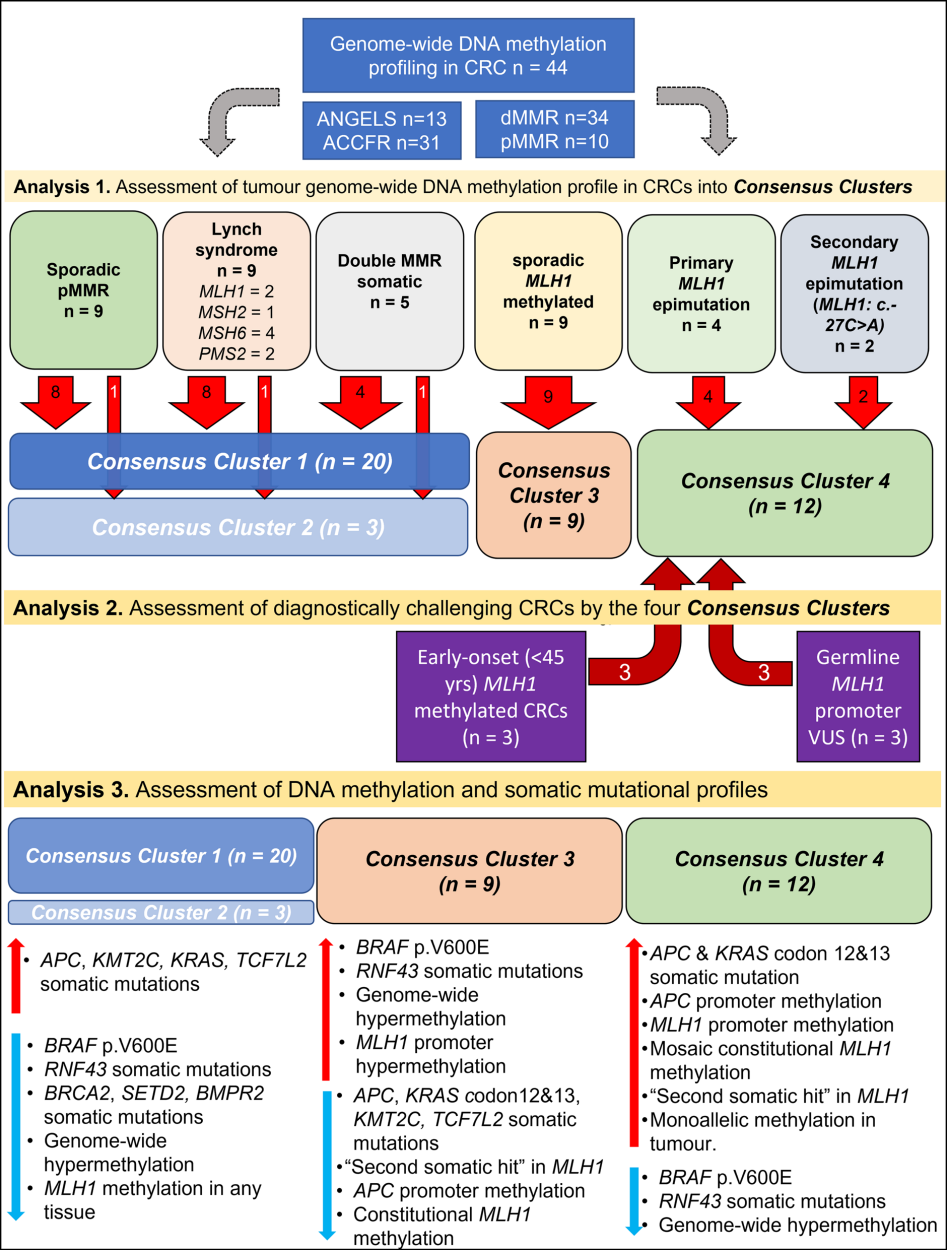
An overview of the study design including descriptions of CRC subgroups and key findings from three main analyses. Analysis 1—genome-wide DNA methylation-based Consensus clustering analysis identified four *Consensus Clusters*. Analysis 2—applying the *Consensus Clustering* to six diagnostically challenging CRCs and the classification of three *MLH1* methylated EOCRCs and three *MLH1* promoter germline VUS carriers into *Consensus Cluster* 4. Analysis 3—further assessment of DNA methylation and somatic mutational profiles associated with each *Consensus Cluster*

We assessed genome-wide DNA methylation and somatic mutational profiles in 44 tumours and matched 14 normal colonic mucosa DNA samples from 43 participants with CRC (Fig. 1). All normal mucosa samples tested were from the surgical specimen from the furthest site of resection from the tumour (i.e. resection margin). Study participants were selected from the ANGELS study [27] or from the Australasian Colon Cancer Family Registry [28]. Immunohistochemical staining (IHC) for expression of the four MMR proteins (MLH1, MSH2, MSH6 and PMS2) was performed on each CRC using previously published protocols [29]. Tumour *MLH1* gene promoter hypermethylation was tested using two locus-specific detection techniques, MethyLight [30] and methylation-sensitive high-resolution melting (MS-HRM) [31]. Tumours showing > 10% methylation by MethyLight and > 5% by MS-HRM were considered positive for *MLH1* promoter methylation and further tested for *MLH1* methylation in blood-derived DNA to identify *MLH1* epimutation. For each of the 43 participants included in the study, the MMR genes, including the *MLH1* gene promoter, were screened to identify germline pathogenic variants as previously described [29] or from multigene panel testing as part of the clinical management. Participants with *MLH1* promoter hypermethylation (> 10%) in blood but without a germline pathogenic variant were classified as a primary *MLH1* epimutation. Of 44 CRCs, 41 tumour DNA and matched blood-derived DNA were also sequenced using whole exome sequencing (WES; *n* = 27) [27] or by a custom designed 298 gene panel sequencing (Panel; *n* = 14) [32].

Thirty-eight CRCs from 37 participants that were classified into six confirmed CRC subtypes were used as reference groups (Fig. 1):

1. “*LS-CRCs*”—MMR-deficient CRCs from participants with LS including 2 × *MLH1,* 2 × *MSH2*, 3 × *MSH6* and 2 × *PMS2* germline pathogenic variant carriers with no *MLH1* promoter hypermethylation in the tumour and blood-derived DNA (n = 9 CRCs from nine participants).
2. “*Sporadic MLH1 methylated CRCs*”—CRCs showing loss of MLH1/PMS2 by IHC with *MLH1* promoter hypermethylation in the tumour but absent in the blood and/or normal mucosa-derived DNA and no germline MMR gene pathogenic variants identified (*n* = 9 CRCs from nine participants).
3. “*Primary MLH1 epimutation CRCs*”—CRCs showing loss of MLH1/PMS2 by IHC resulting from primary *MLH1* epimutation in the absence of *MLH1* promoter *cis*-variants with *MLH1* promoter hypermethylation in the tumour and blood-derived DNA and no germline MMR gene pathogenic variants identified (*n* = 4 CRCs from three participants). One normal mucosa DNA was included to assess the constitutional nature of *MLH1* methylation.
4. “*Secondary MLH1 epimutation CRCs*”—CRCs showing loss of MLH1/PMS2 by IHC resulting from a secondary *MLH1* epimutation (*MLH1*: c.-27C > A), demonstrating *MLH1* promoter hypermethylation in the tumour and blood-derived DNA (*n* = 2 CRCs from two participants). One normal mucosa DNA was included.
5. “*Double MMR somatic CRCs*”—MMR-deficient CRCs with two somatic mutations in the MMR gene indicated as defective by the pattern of protein loss by IHC and no *MLH1* promoter hypermethylation in tumour and blood-derived DNA and no germline MMR gene pathogenic variants identified (*n* = 5 CRCs from five participants).
6. “*MMR-proficient CRCs*”—CRCs with retained/normal expression of all four MMR proteins by IHC and absence of *MLH1* promoter hypermethylation in tumour and blood-derived DNA and no germline pathogenic variants (*n* = 9 CRCs from nine participants).

In addition to the six reference CRC subtypes, we tested two groups of six diagnostically challenging CRCs (Fig. 1):

1. “*MLH1 promoter VUS CRCs*”—Carriers of *MLH1* promoter VUS including two carriers of germline *MLH1:* c.-11C > T and one carrier of germline *MLH1*: c.-[28A > G; 7C > T] *in cis* (*n* = 3 CRCs from three participants). Tumour *MLH1* methylation was tested by two loci-specific techniques described above. No blood methylation was detected by the clinical testing methodology (i.e. MS-MLPA). All cases had no reported CRCs in the first-degree relatives.
2. “*MLH1 methylated early-onset CRCs (EOCRCs)*”— CRCs showing loss of MLH1/PMS2 by IHC with *MLH1* promoter methylation in tumour and CRC diagnosis < 45 years and no germline MMR pathogenic variants or double somatic MMR gene mutations (*n* = 3 CRCs from three participants). No blood methylation was detected by the clinical testing methodology (i.e. MS-MLPA). All cases had no reported CRCs in the first- and second-degree relatives.

### DNA methylation array processing

Tumour and normal mucosa DNA were isolated from macro-dissected formalin-fixed paraffin-embedded (FFPE) specimens using the QIAmp DNA FFPE Tissue Kit (Qiagen, Hilden, Germany). Genomic DNA was bisulphite converted and restored as previously described [33, 34]. Tumour and normal mucosa DNA methylomes were profiled using the Infinium HumanMethylation EPIC platform (HMEPIC, Illumina, San Diego, United States) by the Australian Genome Research Facility (AGRF, Melbourne, Australia). Raw data were imported into the R programming software environment (v3.3.2) and processed using the *minfi* Bioconductor package (v1.38.0) [35]. The data underwent *Functional normalisation* [36] with *noob* background correction [37]. *β*-values were used for presenting the data and *M*-values were used for all statistical analyses [38]. Probes with detection *P*-values greater than 0.05 and probes on sex chromosomes were removed from all analyses. Methylation levels were measured from 771,234 probes in total.

### Bioinformatic analysis

Forty-two CpGs overlapping the CpG island (hg19 chr3: 37033539–37036377) associated with the *MLH1* promoter (NM_000249.3) were used for illustrating *MLH1* promoter methylation. Of these, the mean methylation level was calculated across the four CpGs (cg23658326, cg11600697, cg21490561, cg00893636) overlapping the regulatory “C” region [39] and used to determine the *MLH1* promoter methylation status. DNA samples with mean methylation (*β*-values) > 0.2 were considered *MLH1* methylation positive. CIMP status was determined by assessing mean methylation levels across Infinium HMEPIC CpG probes overlapping or nearby five previously described gene promoter regions [40] (*CACNA1G*: cg18337803, cg20467136, cg23614229, cg11262815; *RUNX3:* cg06377278, cg27095256; *SOCS1*: cg06220235; *NEUROG1*: cg04620091; and *IGF2:* cg16977706). Samples with methylation (> 0.2) at 3 or more of these 5 gene regions were considered CIMP-high. Differentially methylated regions (DMRs) analysis was performed using “DMRcate” package (v2.6.0) [41].

The *Consensus Cluster* analysis was performed using “ConsensusClusterPlus” package (v1.56.0) [42] on the 77,113 most variably methylated CpGs between the 38 CRCs from the six reference groups ranked by standard deviation (SD). These probes constituted 10% of all CpG probes. The *Consensus Cluster* analysis was performed using the default setting and four total clusters (*k’*s) were selected after we found that testing for > 4 clusters provided no additional clusters from our 38 reference CRC samples. The “consensus class assignments” were used to define the sample classification. A principal component analysis (PCA) was performed to test the validity of the observed *Consensus Clusters*.

### Tumour sequencing

Twenty-seven CRCs were sequenced by WES and 14 CRCs were sequenced by the targeted multigene panel. Three CRCs (1 LS-CRC, 1 sporadic *MLH1* methylated, 1 primary *MLH1* epimutation) were excluded from the methylation profiling due to insufficient tumour DNA material remaining for testing. Peripheral blood-derived DNA was extracted using the DNeasy blood and tissue kit (Qiagen) and sequenced as germline references. For WES capture, the Clinical Research Exome V2 kit (Agilent Technologies, Santa Clara, United States) was performed at AGRF as previously described [27].

Adaptor sequences were trimmed using *trimmomatic* v0.38 [43] and aligned to the GRCh37 human reference genome using the BWA (v.0.7.12). Germline variants were called using HaplotypeCaller (GATK library v.4.0.0, Broad Institute). Somatic single nucleotide variants (SNVs) and insertions/deletions (INDELs) were called using Strelka (v.2.9.2) [44] . For both WES and panel, variants were filtered for PASS called by Strelka with a minimum variant allele fraction (VAF) of 0.04 and a minimum coverage depth of 30× for tumour analyses. For consistency, non-overlapping regions between the WES and panel captures were removed, except for the *MLH1* promoter region. WES and panel sequencing was used to identify germline variants across the *MLH1* promoter region up to 1500 base pairs (bp) for the panel sequencing and 2125 bp for WES from the transcription start site. Tumour microsatellite instability (MSI) status was determined bioinformatically using MANTIS [45] with a cut-off for high levels of MSI (MSI-H) of ≥ 0.245 for WES and a cut-off of MSI-H of ≥ 0.252 for panel sequenced tumours [32]. Loss of heterozygosity (LOH) of *MLH1* was determined using LOHdeTerminator v0.5 (https://github.com/supernifty/LOHdeTerminator) by assessing regions of the genome containing heterozygous germline variants that appear to be either homozygous reference or homozygous alternative in the somatic sample, based on an allele frequency range of 0.3 to 0.7 in the germline variant and a difference of greater than 0.3 in the somatic variant.

The *maftools* (v2.12.0) Bioconductor package was used for analysing and visualising somatic variants [46]. Unless described otherwise, *P-*values were derived from Fisher’s exact tests. The list of 32 genes that undergo frequent somatic mutations was retrieved from TCGA COAD samples [47]. Of those, 15 and 17 genes were identified from hypermutated (described as having a high TMB (10–100 mutations/megabase)) CRCs and non-hypermutated (TMB < 10 mutations/megabase) CRCs, respectively [47]. *APC* and *TCFL2* overlapped in both lists and four genes (*TTN, FAM123B*, *KIAA1804*, *EDNRB*) were not captured by the panel sequencing used in this study. We also assessed somatic mutations in four commonly mutated genes (*AXIN2*, *CCND1*, *ZNRF3*, *RNF43*) associated with the *Wnt* pathway [48] as well as two DNA polymerase genes (*POLE*, *POLD1*) [49], and five genes (*DNMT1*, *TET1*, *TET2*, *TET3*, *MBD4*) related to DNA methylation machinery [50].

### *MLH1* promoter methylation detection using droplet digital PCR (ddPCR)

Twenty nanograms of bisulphite-modified blood, normal mucosa and buccal/saliva-derived DNA were tested using the Bio-Rad QX200 ddPCR system (Pleasanton, USA) with the ddPCR Supermix for Probes (no dUTP) (Bio-Rad) and the inclusion of 0.1X Q Solution (Qiagen), 800 nM of each primer and 400 nM of each probe (Bioneer Pacific, Daejeon, South Korea). Sequences for primers and probes are shown in Additional file 1: Table S1.

### Detecting allelic MLH1 methylation using methylation-specific PCR and pyrosequencing

SMART-MSP (Sensitive Melting Analysis after Real-time Methylation-Specific PCR) reactions were performed in technical duplicates on a Mic qPCR Cycler (BMS, Sydney, Australia) as previously described [51] to specifically amplify only methylated epialleles. The primer sequences (Bioneer) can be found in Additional file 1: Table S1. The amplified methylated epialleles were then pyrosequenced on a Qseq instrument (BMS) using the Q48 Advanced CpG kit (Qiagen) and Streptavidin Mag Sepharose beads (Cytiva, MA, USA) to assess the genotypes of the SNPs on only the methylated epialleles. The pyrosequencing data were analysed with Qseq software 2.4.4 (BMS).

## Results

### The CRC subgroups tumour characteristics

The characteristics of the participants and their CRCs by subtype are described in Additional file 1: Tables S2 and S3. The HMEPIC-based DNA methylation levels (β-value) for each of the CRC and normal mucosa samples across the *MLH1* promoter are shown in Additional file 2: Fig. S1 and Additional file 1: Table S3. CRC tumour samples from the primary (mean *β* = 0.77 ± 0.06 SD) and secondary (0.74 ± 0.16) *MLH1* epimutation carriers showed *MLH1* methylation levels (i.e. hypermethylation) consistent with tumour samples from people with sporadic *MLH1* methylated CRCs (0.55 ± 0.09). For the normal mucosa samples, *MLH1* promoter methylation was observed at high levels in only the primary (mean *β*-value = 0.40) and secondary (*β* = 0.39) *MLH1* epimutation CRC groups. Each of the three *MLH1* methylated EOCRCs from the diagnostically challenging group showed *MLH1* promoter hypermethylation in their tumours (*β* = 0.34, 0.42 and 0.67) but not in the single normal mucosa sample tested (*β* = 0.07). Similarly, both the CRCs from the *MLH1:* c.-11C > T VUS carriers showed *MLH1* promoter hypermethylation (*β* = 0.43 and 0.39, respectively), while the CRC from the *MLH1*: c.-[28A > G; 7C > T] VUS carrier was only moderately increased (*β* = 0.14), compared with their respective normal mucosa samples (*β* = 0.06, 0.1 and 0.09, respectively) but not meeting our threshold of 0.2 (Additional file 2: Fig. S1).

We assessed differential patterns of somatic mutations in key CRC genes between the six reference groups and the two groups of diagnostically challenging CRCs (Additional file 3: Fig. S2). *MLH1* methylated CRCs were associated with less frequent mutations in *APC*, *KRAS* and *TCF7L2*, and frequent mutations in *BRAF* p.V600E and *RNF43* (*P* < 0.05). Consistent with LS-CRCs, all *MLH1* epimutation, *MLH1* promoter VUS CRCs and *MLH1* methylated EOCRCs carried at least *APC* or *TCF7L2* somatic mutations and frequent *KRAS* codon 12&13 mutations (Additional file 1: Table S4). Unlike the sporadic *MLH1* methylated CRCs, these groups’ CRCs also lacked the *BRAF* p.V600E somatic mutation (*P* < 0.01) and had less frequent *RNF43* somatic mutations (*P* = 0.04). Of the five genes of the epigenetic machinery, only *TET2* somatic mutations showed a significant enrichment in LS-CRC (*P* < 0.01; Additional file 1: Table S4).

### Genome-wide DNA methylation consensus clusters distinguish *MLH1* epimutation carriers CRCs from the other reference group CRCs

The genome-wide DNA methylation profiles were compared between 38 CRCs from the six reference tumour groups using a consensus clustering analysis based on the 77,113 most variably methylated CpGs. The consensus values are illustrated in a heatmap (Additional file 4: Fig. S3A), and the raw values are provided in Additional file 12: data. Six CRCs from two diagnostically challenging CRC groups (*MLH* promoter VUS CRCs and *MLH1* methylated EOCRCs) were excluded in this training analysis. The analysis identified four *Consensus Clusters* (Table 1).

- *Consensus Cluster 1*—comprised 20 CRCs (8/9 of LS-CRCs, 8/9 of the MMR-proficient CRCs and 4/5 of double MMR somatic CRCs). This cluster had the youngest mean age of CRC diagnosis (38.7 ± 10.6 interquartile range, IQR), the lowest level of *MLH1* promoter methylation (mean β=0.08) and the lowest overall methylation levels across the variably methylated CpGs (mean *β* = 0.32).
- *Consensus Cluster 2*—comprised three CRCs (1 LS-CRC, 1 MMR-proficient and 1 double MMR somatic CRCs) and was also characterised by low *MLH1* promoter methylation (mean β=0.08). The double MMR somatic CRC (diagnosis age = 57 years) was also CIMP-high, though having low *MLH1* promoter methylation. All samples from *Consensus Cluster 2* had higher overall methylation (mean *β *= 0.42) across the variably methylated CpGs when compared with *Consensus Cluster 1* group (*P *= 0.02).
- *Consensus Cluster 3*—consisted of all nine sporadic *MLH1* methylated CRCs and had the oldest mean age at CRC diagnosis (62.5 years ± 10.2 IQR). This cluster demonstrated the highest overall methylation across the variably methylated CpGs (mean *β* = 0.45).
- *Consensus Cluster* 4—comprised all six primary and secondary *MLH1* epimutation carrier CRCs. Tumours in this cluster demonstrated low overall methylation levels (mean *β* = 0.35) across the variably methylated CpGs similar to *Consensus Cluster 1*.

**Table 1:** Overview of the sample composition and tumour characteristics within each of the four *Consensus Clusters* derived from genome-wide DNA methylation profiling of six CRC subtypes (reference groups)

	Consensus cluster 1	Consensus cluster 2	Consensus cluster 3	Consensus cluster 4
Number of CRCs	20	3	9	6
Age at CRC diagnosis (mean ± s.d)	38.7 ± 10.6	45.9 ± 12.8	65.5 ± 9.65	39.2 ± 11.3
*MLH1* promoter methylation^a^ (mean ± s.d)	0.076 ± 0.02	0.082 ± 0.02	0.548 ± 0.089	0.761 ± 0.045
CIMP^b^ (% positive)	0 (0%)	1 (33%)	9 (100%)	0 (0%)
Mean methylation across the VM-CpGs^c^	0.32	0.42	0.45	0.35
Lynch syndrome (*n* = 9)	8	1	0	0
MMR-proficient CRC (*n* = 9)	8	1	0	0
Double somatic MMR mutation CRC (*n* = 5)	4	1	0	0
Sporadic *MLH1* methylated CRCs (*n* = 9)	0	0	9	0
*MLH1* primary epimutation (*n* = 5)	0	0	0	4
*MLH1* secondary epimutation (*n* = 1)	0	0	0	2

^a^Mean methylation (*β*-values) across the regulatory C region of *MLH1*

^b^CIMP was determined by the methylation levels of CpG probes overlapping five previously defined genes [40]

^c^Mean methylation levels across the 77,113 most variably methylated CpGs (VM-CpGs), which was used for defining the *Consensus Clusters*. s.d.—standard deviation, VM-CpGs—(77,113) variably methylated CpGs defined by having high variation in the methylation patterns as ranked by standard deviation across 38 reference CRCs

**Fig. 2:**
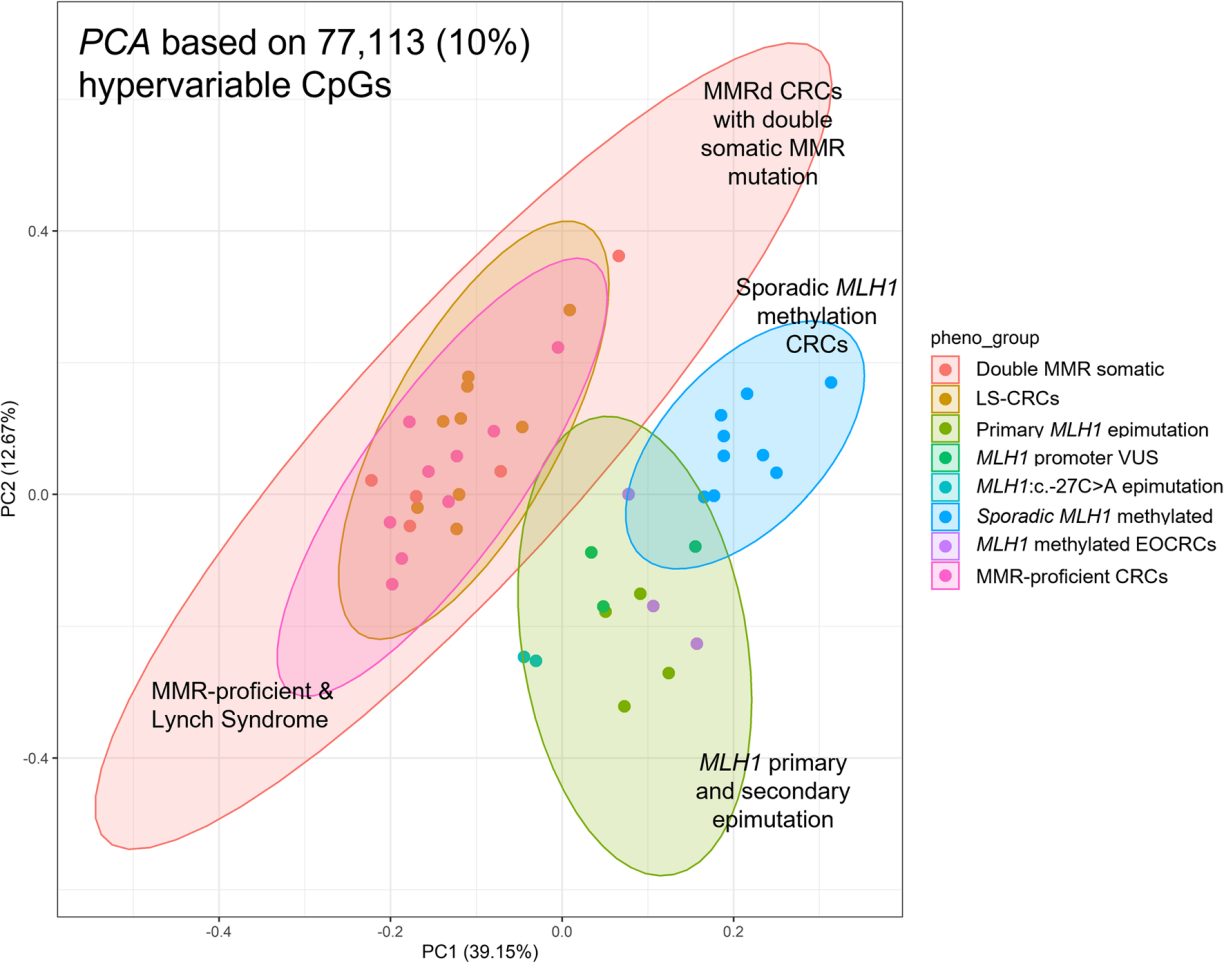
Principal component analysis (PCA) showing genome-wide DNA methylation similarities between individual tumour, based on 77,113 variably methylated (VM)-CpG probes. Tumour samples of different CRC subgroups are shown in different colours. The *MLH1*-VUS group includes CRCs from 3 *MLH1* germline VUS carriers (two *MLH1*: c.-11C > T and one *MLH1*: c.-[28A > G; 7C > T] carriers)

The PCA analysis of the 77,113 most variably methylated CpGs, applied as an alternate approach to Consensus clustering, demonstrated three distinct groupings related to sporadic *MLH1* methylated CRCs, primary and secondary *MLH1* epimutation CRCs and a third group comprising LS-CRCs, double MMR somatic and MMR-proficient CRCs (Fig. 2) largely reflecting the groupings from the consensus cluster analysis. Additional file 5: Fig. S4 shows overall methylation patterns across the variably methylated CpGs.

### DNA methylation signatures associated with CRCs of MLH1 epimutation carriers

We performed differential methylation analysis between CRCs from primary and secondary (*MLH1*: c.-27C > A) epimutation carriers. A single differentially methylated (*FDR or False Discovery Rate*-adj *P* = 0.0004) CpG probe (cg15103403) was identified, located within the *LRRFIP2* gene (chr3: 37110355). A clear hemi-methylation (~ 50%) pattern was observed in both CRCs from secondary epimutation carriers (Additional file 6: Fig. S5). There were no other CpG probes within the 4 kb flanking region and, therefore, regional methylation differences could not be assessed using the HMEPIC array data.

To further understand the tumour DNA methylation differences between those with sporadic (acquired) versus constitutional *MLH1* promoter hypermethylation, we compared the genome-wide DNA methylation profiles of sporadic *MLH1* methylated CRCs (*n* = 9) with those from *MLH1* epimutation CRCs (*n* = 6) to identify DMRs. Given the paucity of differentially methylated CpGs sites between the primary and secondary *MLH1* epimutation CRCs shown above, these two groups were combined as the *MLH1* epimutation group. This identified 1447 DMRs (FDR-adj *P* < 0.01 & mean absolute *β* differences > 0.2) where 99% (1438/1447) of these DMRs were hypermethylated in the sporadic *MLH1* methylated CRC group when compared with the epimutation group. In 9 (1%) of the DMRs, the mean methylation was greater by > 0.2 (*β*) in the *MLH1* epimutation group and included the *APC, MAD1L1, YPEL2, CRTC1, SSBP3, STARD13* genes and a non-coding RNA *KLRK1-AS1* loci (Additional file 1: Table S5).

**Fig. 3:**
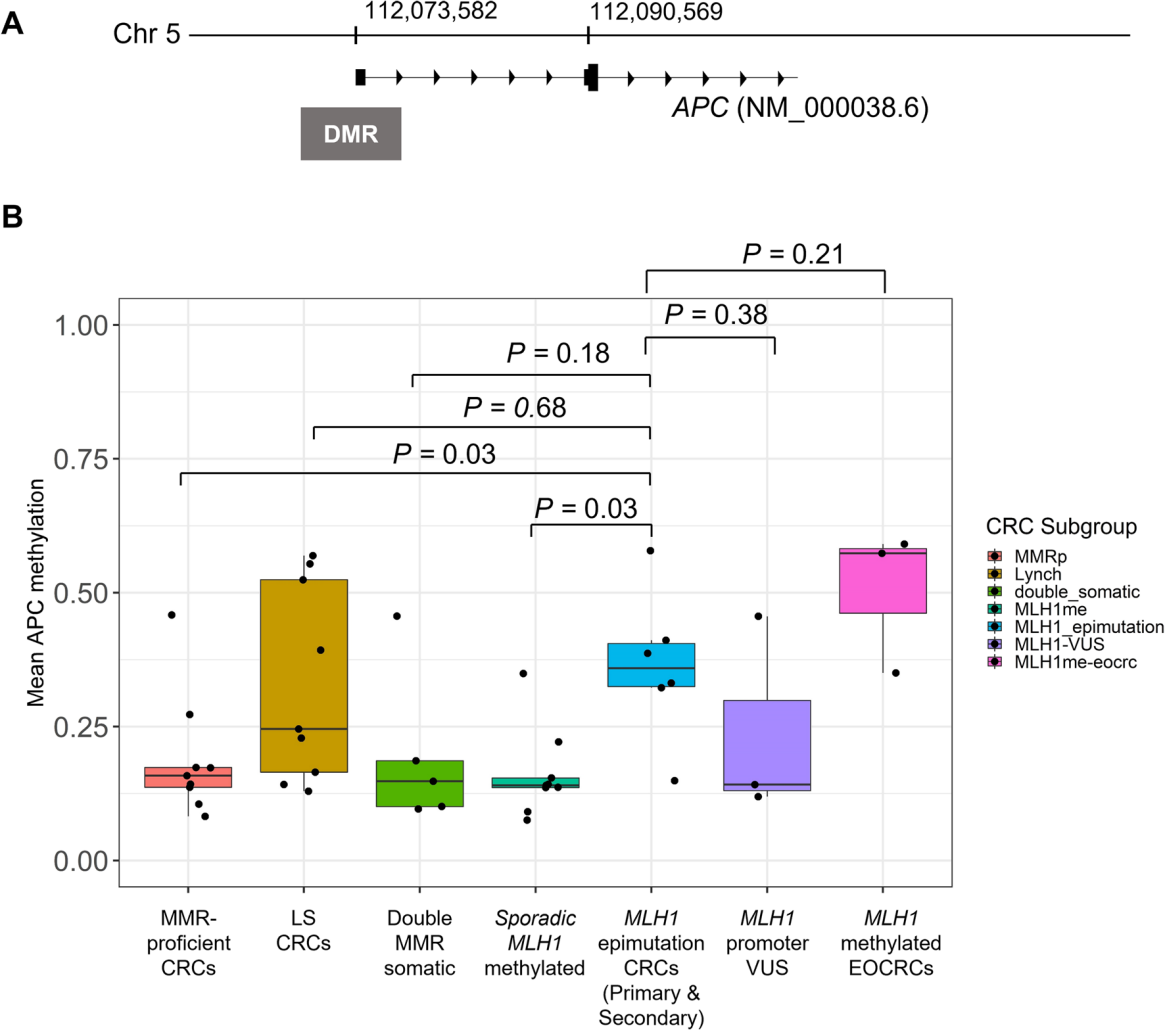
DNA methylation patterns across the DMR (chr5: 112,072,926–112,073,958) overlapping the *APC* gene. **A**. The DMR (differentially methylated region) associated with the *MLH1* epimutation CRCs. **B**. Bar plot showing mean DNA methylation levels by CRC subgroups. Error bars denote standard deviation. *MLH1* epimutation group includes primary *MLH1* epimutation and secondary *MLH1* epimutation (c.-27C > A) CRCs. *MLH1* promoter VUS group includes CRCs from two *MLH1*: c.-11C > T and one *MLH1*: c.-[28A > G; 7C > T] carriers

Of these seven genes, *APC,* a known driver of CRC tumourigenesis [52] , showed the most significant differences (Stouffer transformed *P* = 1.5 × 10^–10^) between the two groups. The *APC* promoter region (chr5: 112072926–112073958) showed higher methylation levels in the *MLH1* epimutation group (mean *β* = 0.36 ± 0.14 SD) when compared with sporadic *MLH1* promoter methylated CRCs (0.16 ± 0.08; *P* = 0.03) and when compared with the MMR-proficient CRCs (0.19 ± 0.18; *P* = 0.03), but were not different to the LS-CRCs (0.33 ± 0.11) or double MMR somatic CRCs (0.20 ± 0.15) (Fig. 3). The *APC* promoter hypermethylation (mean *β* > 0.2) was detected in only one of the five double MMR somatic CRCs. Both the unique and common DMRs to each reference group are shown in Additional file 7: Fig. S6.

### Genome-wide DNA methylation Consensus Clustering for categorising carriers of *MLH1* promoter VUS and *MLH1* methylated EOCRCs

We applied the *Consensus Cluster* approach using the same 77,113 variably methylated CpGs to *MLH1* promoter VUS CRCs (*n* = 3) and *MLH1* methylated EOCRCs (*n* = 3). The consensus values are illustrated in Additional file 4: Fig. S3B. The pedigrees for each case are shown in Additional file 8: Fig. S7. The two CRCs from *MLH1*: c.-11C > T and CRC from *MLH1*: c.-[28A > G; 7C > T] fitted to *Consensus Cluster 4*. Similarly, the three *MLH1* methylated EOCRCs also fitted to *Consensus Cluster 4*, though no candidate germline *cis* variants or VUS were found in the *MLH1* promoter region for these three participants.

### CRCs from *MLH1*: c.-11C > T VUS and *MLH1* methylated EOCRCs show tumour characteristics similar to known *MLH1* epimutation CRCs and demonstrate mosaic monoallelic MLH1 epimutation patterns

CRCs from *MLH1*: c.-11C > T VUS carriers had late diagnosis age, demonstrated loss of MLH1/PMS2 protein expression by IHC and showed *MLH1* hypermethylation in the tumour concordantly by both loci-specific techniques and the HMEPIC data (Table 2). One of the *MLH1*: c.-11C > T VUS carriers demonstrated 29% methylation in the *MLH1* gene promoter in a metachronous duodenal cancer and showed 1% *MLH1* methylation in the blood-derived DNA as detected by MethyLight. The second *MLH1*: c.-11C > T VUS carrier demonstrated 0% *MLH1* methylation in their blood-derived DNA by MethyLight. CRCs from both *MLH1:* c.-11C > T VUS carriers showed a “second somatic hit” in *MLH1* as a single nucleotide variant or LOH (large deletion of wildtype allele, Additional file 9: Fig. S8A). Of note, a “second somatic hit” by LOH or single nucleotide variant in *MLH1* was also observed in each of the primary and secondary *MLH1* epimutation CRCs and in 6/8 (75%) of LS-CRCs but no second somatic hit in *MLH1* was observed in the sporadic *MLH1* methylated CRCs.

**Table 2:** The characteristics of the six CRCs in the diagnostically challenging group comprised of carriers of *MLH1* promoter VUS (*n* = 3) and *MLH1* methylated early-onset CRCs (*n* = 3)

Inclusion Group	Dx age (yrs)	Sex	CRC anatomical site	MMR IHC	MSI^e^	Mean methylation in the VM-CpGs^d^	CIMP in CRC (Total positive)^b^	Mean MLH1 promoter methylation (Tumour) ^a^	Blood *MLH1* promoter methylation (MethyLight)^c^	Non-tumour *MLH1* promoter methylation (ddPCR)^d^	Additional tissue tested (MethyLight)^c^	*MMR genes* LOH/somatic mutation	*APC* promoter methylation^f^	*KRAS somatic mutation*
*MLH1* VUS (*MLH1*: c.-11C > T)	68	F	Proximal	MLH1/PMS2 loss	MSI-H	0.288	Negative (0/5)	0.43	0%	Blood—0% NMadj—2.3% NMdist—1.2% Buccal—6.5%	–	*MLH1* LOH^g^	0.12	None detected
*MLH1* VUS (*MLH1*: c.-11C > T)	60	M	Proximal	MLH1/PMS2 loss	MSI-H	0.392	Negative (2/5)	0.39	1%	Blood—4.5% NMadj—3.4% NMdist—nt Buccal—13.9%	Duodenal cancer—29%	*MLH1*:p.S131Ter	0.46	p.G12D
*MLH1* VUS *MLH1:* *c-[28*A > G*; 7*C > T]	35	F	Proximal	MMRp (Incomplete loss of MLH1/PMS2)	MSS	0.282	Negative (1/5)	0.14	0%	Blood—0% NMadj—0% NMdist—0% Buccal—0%	–	None detected	0.14	p.G12A
*MLH1me EOCRC*	37	F	Proximal	MLH1/PMS2 loss	MSI-H	0.374	Negative (2/5)	0.34	0%	Blood—0% NMadj—nt NMdist—0% Buccal—nt	–	*MLH1*: p.S108AfsTer28	0.59	p.G12D
*MLH1me EOCRC*	43	M	Proximal	MLH1/PMS2 loss	MSI-H	0.338	Negative (0/5)	0.42	1%	Blood—< 1% NMadj—nt NMdist—4.1% Buccal—2.4%	TVA—49%	*MLH1*: p.X701_splice	0.57	None detected
*MLH1me EOCRC*	31	M	Proximal	MLH1/PMS2 loss	MSI-H	0.353	Negative (1/5)	0.67	0%	Blood—0% NMadj—nt NMdist—nt Buccal—0%	–	*MLH1* LOH^g^	0.35	None detected

*NM*_*dist*_ denotes distal normal mucosal samples derived from resection margin (i.e. non-adjacent), *NM*_*adj*_ denotes normal mucosal samples derived from histologically normal mucosal samples adjacent to the tumour, *TVA* Tubulovillous Adenoma

^a^The values indicate mean *β*-values estimated from 4 CpGs overlapping *MLH1* promoter C region [39] derived from the HMEPIC data

^b^As assessed from the HMEPIC

^c^As assessed by MethyLight technique

^d^Mean methylation levels across the 77,113 most variably methylated CpGs, which was used for defining the *Consensus clusters*

^e^Estimated using MANTIS

^f^Mean methylation from 16 CpGs across the *APC* promoter c(hr3:112,072,926–11,207,395)

^g^LOH—loss of heterogygosity (across the *MLH1* locus). The LOH plot is shown in Additional file 9: Fig. S8

The CRC from the carrier of *in cis* variants *MLH1*: c.-[28A > G; 7C > T] was diagnosed at 35 years of age, showed “patchy” loss of MLH1/PMS2 by IHC in both tumour and adjacent normal cells, with mean *β* of 0.14 in *MLH1* promoter (Table 2 and Additional file 2: Fig. S1C). For this CRC, no “second somatic hit” in *MLH1* was observed.

The three *MLH1* methylated EOCRCs each showed loss of MLH1/PMS2 protein expression by IHC, showed high levels of *MLH1* methylation in their tumours (*β* = 0.34, 0.42, 0.67) and did not have the *BRAF* p.V600E mutation or CIMP-high (Table 2 and Additional file 3: Fig. S2). One of the three demonstrated 1% *MLH1* methylation in the blood-derived DNA by MethyLight and was additionally found to have *MLH1* methylation (49%) in a conventional tubulovillous adenoma contiguous to the CRC. Consistent with constitutional *MLH1* epimutation CRCs, all three EOCRC *MLH1* methylated tumours showed a second somatic hit in *MLH1* (Additional file 9: Fig. S8B). Tumour hypermethylation (~ 50%) of the *APC* promoter region was present in all three *MLH1* methylated EOCRCs similar to *MLH1* epimutation CRCs (mean *β* = 0.57) and LS-CRCs (0.33) but higher than the MMR-proficient (0.19) and sporadic *MLH1* methylated CRCs (0.16).

To further investigate potentially low-level mosaic constitutional *MLH1* methylation, we employed methylation-sensitive ddPCR to measure *MLH1* promoter methylation in blood, normal mucosa and buccal-derived DNA. Mosaic constitutional *MLH1* methylation was confirmed in both *MLH1*: c.-11C > T VUS carriers present in low levels (1.6%-13.4%) across the three tissue types (Table 2 and Additional file 1: Table S6). Both carriers showed positive (> 1%) methylation in normal mucosa and buccal DNA samples, whilst one also showed *MLH1* methylation in blood DNA samples. One of the three *MLH1* methylated EOCRC cases showed a similar mosaic methylation pattern in all three tissue samples. In comparison, the three primary and two secondary *MLH1* epimutation cases all showed *MLH1* hypermethylation in blood and normal mucosa-derived DNA samples, whilst CRCs from other reference groups had no detectible methylation in blood or normal mucosa except for one sporadic *MLH1* methylated CRC (age of CRC diagnosis = 78 years), which showed 3.8% methylation only in the distant normal mucosa but not in blood or adjacent normal mucosa. The *MLH1*: c.-[28A > G; 7C > T] VUS carrier did not show evidence of *MLH1* methylation in blood, normal mucosa or buccal DNA samples. The ddPCR results are described in Additional file 1: Table S6 and also illustrated in Additional file 10: Fig. S9 for representative samples.

Using the SMART-PCR and pyrosequencing, we tested for allelic methylation levels in tumour DNA samples from two *MLH1:* c.-11C > T VUS carriers and one *MLH1* methylated EOCRC case with a nearby single nucleotide polymorphism (SNP) (c.-93G > A/rs1800734). We found monoallelic *MLH1* methylation in tumours from both *MLH1*: c.-11C > T VUS carriers where the allele carrying the c.-11C > T variant was methylated (Additional file 11: Fig. S10A & B). Here, as the *MLH1*: c.-11C reference residue is affected by the sodium bisulphite treatment, the methylation-specific PCR was targeted to the antisense strand (G > A). In two sporadic *MLH1* methylated CRCs without the c.-11C > T (G > A antisense) variant (Additional file 11: Fig. S10C, D), the G (reference) allele showed sole amplification in absence of the A variant allele. In the *MLH1* methylated EOCRC case with the heterozygous *MLH1*: c.-93G > A SNP, methylation was specifically associated with the SNP (A) allele showing sole amplification of the A allele (Additional file 11: Fig. S10E). In comparison, biallelic methylation was confirmed in all sporadic *MLH1* methylated CRCs with methylation present in both alleles with *MLH1:* c.93A or G SNP showing equal amplification of both A and G alleles (Additional file 11: Fig. S10 F, G).

### The somatic mutation profiles differ between the four methylation-derived consensus clusters

**Fig. 4:**
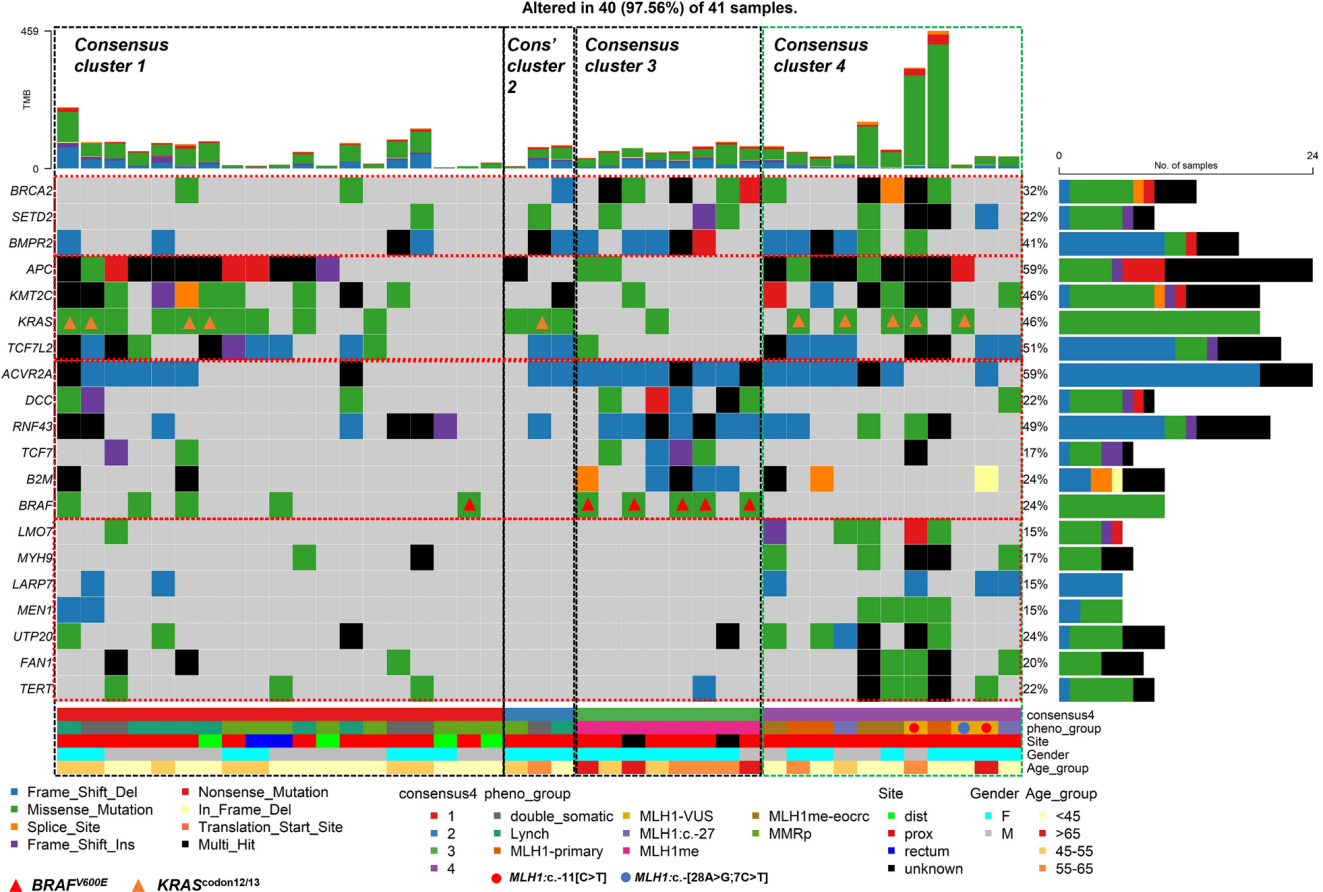
Somatic mutational “oncoplot” showing differential mutational patterns by *Consensus Clusters* as defined by genome-wide DNA methylation. Somatic mutational profiles of 20 novel genes, including 3 genes associated with *Consensus Cluster* 3, 10 genes associated with *Consensus Cluster* 3 and 7 genes associated with *Consensus Cluster* 4 are shown. The samples are shown in vertical lines and ordered by CRC subgroup and their *Consensus Clusters*. The total mutational burden (TMB) shows the accumulative numbers of somatic mutations identified in each tumour sample. Individual tumour samples are further annotated by CRC subgroup, anatomical location, gender and CRC diagnosis age (years). The different types of somatic mutations are shown in different colours, and the compositional barplots illustrate the total loads of somatic mutations separated by the nucleotide changes

We investigated differences in the somatic mutational profiles between the four *Consensus Clusters*. An enrichment analysis identified 20 genes in which somatic mutations were significantly (*P* < 0.05) associated with each of the *Consensus Clusters* (Fig. 4). Specifically, six genes (*ACVR2A, DCC, RNF43, TCF7, B2M and BRAF)* were associated (*P* < 0.05) with *Consensus Cluster 3* CRCs. Additionally, *Consensus Cluster 3* CRCs (*P* < 0.05) were also associated with less frequent mutations in four genes (*APC, KMT2C, KRAS and TCFL2*) when compared to the rest. *Consensus Cluster 1* CRCs were associated (*P* < 0.05) with infrequent mutations in three genes (*BRCA2*, *SETD2* and *BMPR2*) but no frequently mutated genes were identified. *Consensus Cluster 4* CRCs were associated with frequent somatic mutations in seven genes (*LMO7, MYH9*, *UTP20*, *FAN1*, *LARP7*, *MEN1* and *TERT*) (*P* < 0.05). *MLH1* promoter VUS CRCs and *MLH1* methylated EOCRCs clustered synonymously with *MLH1* epimutation CRCs, showing infrequent mutations in *DCC, RNF43, TCF7, B2M* and *BRAF* but showing frequent mutations in *APC, KMT2C, KRAS* and *TCF7L2*.

## Discussion

This integrative analysis of genome-wide DNA methylation and somatic mutational profiles of 38 CRCs of six clinically relevant subgroups of sporadic and inherited CRCs provides insight into these tumours’ molecular heterogeneity. This study identified unique genome-wide DNA methylation aberrations and somatic mutations associated with rare *MLH1* epimutation CRCs and assessed CRC aetiologies in three germline carriers of *MLH1* promoter VUS and three EOCRCs with *MLH1* methylation (Fig. 1). Unique tumour features were identified that may augment the detection of *MLH1* epimutation carriers, including genome-wide DNA methylation as depicted by *Consensus Clusters*, frequent somatic mutations in *APC*, *KRAS* codons 12&13, *KMT2C* and *TCF7L2*, a second somatic hit in *MLH1* with monoallelic methylation [12], and *APC* promoter methylation.

This study identified mosaic constitutional *MLH1* epimutation associated with the *MLH1*: c.-11C > T germline VUS in two CRCs, detected in non-tumour DNA samples. Both CRCs showed tumour features concordant with CRCs from known constitutional *MLH1* epimutation cases, suggesting the same CRC aetiology. Using methylation-sensitive ddPCR, we identified low-level methylation in blood, normal mucosa and buccal DNA from *MLH1:* c.-11C > T germline VUS carriers. The absence of both the *BRAF* p.V600E mutation and the widespread hypermethylation across the variably methylated CpGs suggests that these CRCs and the *MLH1* promoter hypermethylation have not arisen through the serrated pathway [53], despite their later age at CRC diagnosis. A previous study showed that this variant induced a significant reduction of the *MLH1* transcription [19], though was unable to detect methylation in the blood DNA. These authors described variable CRC diagnosis age with no remarkable family history associated with the c.-11C > T variant [19], suggesting a lower penetrance of this variant.

No evidence of constitutional *MLH1* methylation was identified in normal mucosa, blood or buccal DNA for the *MLH1*: c.-[28A > G; 7C > T] *in cis* germline variant, despite observing weak (*β* = 0.14) methylation in the tumour DNA, heterogenous loss of MLH1/PMS2 protein expression and some of the tumour features associated with known *MLH1* epimutation carriers. In previous studies, these germline variants have been shown to induce a partial and constitutional reduction of *MLH1* expression, however, without causing significant *MLH1* promoter methylation in tumour or normal tissue [24, 54]. Therefore, the findings from the present study remain inconclusive for determining the mechanism of pathogenesis of these germline variants.

This study showed that constitutional *MLH1* epimutation underlie a subset of EOCRCs with *MLH1* methylation by identifying tumour features associated with *MLH1* epimutation and low-level mosaic *MLH1* methylation in non-tumour DNA. Since none were found to carry germline pathogenic variants or VUS in the promoter region, it suggests a de novo origin of mosaic *MLH1* epimutation. These EOCRCs were *BRAF* wildtype with no presentation of CIMP or widespread genome-wide DNA methylation aberrations, suggesting these CRCs did not develop via the serrated pathway [55, 56]. Whilst no evidence that this was related to secondary epimutation or methylation quantitative trait locus was found, we cannot exclude the possibility that the constitutional *MLH1* methylation was caused by germline and probable de novo*,* structural variants including insertion of repetitive elements [57], or a large inversion or duplication involving the *MLH1* gene region [58], for which our sequencing platform did not provide the resolution to detect. Furthermore, the three EOCRCs did not show a family history of Lynch syndrome spectrum cancers, further supporting the absence of a highly penetrant, inherited *cis-*acting genetic variant in these participants (see Additional file 8: Fig. S7 for pedigrees).

Although triaging CRC cases for *MLH1* epimutation testing varies between clinics, young cases with ostensibly sporadic CRCs with *MLH1* methylation have been recommended to be screened for possible *MLH1* epimutation [10, 26] and our findings support the importance of this. Although the transgenerational heritability of primary *MLH1* epimutation is yet to be completely understood [10], a transmission of mosaic *MLH1* epimutation from an asymptomatic carrier into a full-blown *MLH1* epimutation in the offspring has been reported [22], highlighting the clinical importance of early detection of such cases. This will also help alleviate the subsequent cancer risk by recommending appropriate surveillance.

A significant association between *APC* promoter hypermethylation in the CRCs from constitutional *MLH1* epimutation carriers and the *MLH1* methylated EOCRCs was observed. Though not extensively reported, *APC* methylation has been shown to be inversely correlated with CIMP in *BRAF* wildtype CRCs [59], that is consistent with the findings of the current study. Given its primary role as the main *Wnt* regulator, our finding warrants further investigation into the functional importance of *APC* hypermethylation in *MLH1* epimutation and as an additional feature to distinguish *MLH1* epimutation carriers from sporadic *MLH1* methylated CRCs.

This study had several limitations including the small number of *MLH1* epimutation CRCs, germline *MLH1* promoter VUS and *MLH1* methylated EOCRCs. Despite these being rare subtypes of CRCs, further validation in additional CRCs from these groups is needed to confirm our findings and the likely prevalence of *MLH1* methylation mosaicism in EOCRCs. Further studies will also be needed to identify the mechanism underlying mosaicism of *MLH1* methylated EOCRCs. The ability to differentiate *MLH1* epimutations arising de novo (primary *MLH1* epimutation) from those with a genetic basis (secondary) has implications for the relative testing and clinical management. Although no difference was observed in this study, the identification of genome-wide DNA methylation profiles that differentiate primary and secondary *MLH1* epimutation CRCs may require larger sample sizes and, if present, would indicate the need for further genetic testing such as long-read or RNA-sequencing to identify a causative germline variant.

In our study, transcriptional loss associated with monoallelic *MLH1* methylation in *MLH1*: c.-11C > T VUS CRCs and *MLH1* methylated EOCRCs within the blood was not confirmed by expression studies. However, determining reduced monoallelic expression is not feasible due to the low proportion of *MLH1* methylated alleles (mosaicism) in blood DNA. Detecting small changes in expression resulting from only a few methylated alleles in the background of many non-methylated alleles is not feasible. Transcriptional loss of both alleles within the tumour is confirmed by the loss of MLH1 protein expression determined by IHC where one allele is defective due to hypermethylation and the other allele through a second somatic hit.

## Conclusions

*MLH1* epimutations may account for up to 10% of all CRCs with MLH1 protein loss without germline *MLH1* mutation [26] suggesting that currently, *MLH1* epimutations might be underdiagnosed and consequently the true disease burden caused by *MLH1* epimutation is unknown [15, 60]. Currently, no consensus guidelines for triaging potential *MLH1* epimutation carriers exist. Further, unpredictable transgenerational inheritance patterns and the presence of mosaic patterns seen in the carriers, as well as the lack of sensitive testing tools such as genome-wide methylation or ddPCR as demonstrated in this study, contribute towards the current impediment in identifying *MLH1* epimutation carriers and providing personalised clinical management [10]. Here, our study has provided additional molecular features based on genome-wide DNA methylation and somatic mutational landscapes that may be useful for triaging *MLH1* epimutation carriers and provide supporting evidence for resolving VUS associated with *MLH1* epimutation and identifying potential epimutation carriers among young cancer cases with mosaic constitutional *MLH1* epimutation.

## Supplementary Information

**Additional file 1**. **Table S1**: Sequences of primers included in this study. **Table S2**: The characteristics of 38 CRCs from six reference CRC groups. **Table S3**: The characteristics of 44 tumour and 14 normal colonic mucosal samples from the 43 people included in the study. **Table S4**: Summary of somatic mutations occurrent in key CRC genes and CRC subgroups associated with frequent or less frequent somatic mutation in the described gene. **Table S5**: Differentially Methylated Regions between tumours of MLH1 epimutation carriers and sporadic *MLH1* methylated CRCs. **Table S6**: MLH1 promoter methylation levels in non-tumour DNA samples measured by methylation-sensitive digital droplet PCR.

**Additional file 2**. **Figure S1**:DNA methylation levels across the CpG island associated with the *MLH1* promoter by CRC subgroups: 1) MMR-proficient CRCs, 2) Lynch-syndrome associated CRCs, 3) double MMR somatic mutation CRCs, 4) sporadic *MLH1* methylated CRCs, 5) constitutional primary *MLH1* epimutation CRCs and 6) constitutional secondary *MLH1* epimutation CRCs, as well as two diagnostically challenging CRC groups: 1) *MLH1* promoter VUS carriers with *MLH1*: c.-11C>T and *MLH1*: c.-[28A>G; 7C>T] and 2) *MLH1* methylated EOCRCs. DNA methylation of tumour-derived  DNA are shown in red and normal mucosal  DNA is shown in blue. All normal mucosa samples were from resection margin, not adjacent to the tumour. Yellow boxes denote the regulatory C region as described in Deng et al. Bar plots showing mean methylation within the regulatory C region of *MLH1* gene promoter across all samples for each CRC group. Tumour and normal mucosal samples are separately shown. Error bars denote standard deviation. Close-up view of four CpGs overlapping the regulatory C region.

**Additional file 3**.** Figure S2**: Oncoplot showing somatic mutational profiles at the 26 recurrently mutated CRC genes identified in CRC tumours from TCGA, 4 additional Wnt pathway associated genes and 5 genes related to the DNA methylation machinery. The total mutational burden  shows the accumulative numbers of somatic mutations identified in each tumour sample. Individual tumour samples are further annotated by CRC subgroup, anatomical location, gender and CRC diagnosis age. The different types of somatic mutations are shown in different colours and the compositional barplots illustrate the total loads of somatic mutations separated by the nucleotide changes. One primary *MLH1* epimutation CRC had a *POLE* somatic mutation in the exonuclease domain and also had the highest TMB with 459 total somatic mutations.

**Additional file 4**. **Figure S3**: Heatmaps illustrating the consensus matrix identified by the ConsensuClusterPlus analysis. A illustrates the consensus matrix estimated on 38 reference group CRCs. B illustrates the same analysis but performed on the complete dataset of 44 CRCs including six diagnostically challenging CRCs. The final consensus cluster classification for individual samples are shown in four different colours. The darker heatmaps indicate the stability evidence for classifying individual samples into each cluster. The raw consensus values are provided in Supplementary data.

**Additional file 5**. **Figure S4**: “Circos” plots showing mean genomic hypomethylation and hypermethylation patterns in tumours of five reference CRC groups. For the *MLH1* epimutation group, five primary and two secondary epimutation CRCs were combined in this analysis. Mean methylation values are shown per each CRC group.

**Additional file 6**. **Figure S5**: DNA methylation patterns at CpG site cg15103403 within *LRRFIP2* gene for each of the six reference CRC subgroups and the two diagnostically challenging CRC subgroups.

**Additional file 7**. **Figure S6**: Venn diagrams showing numbers of Differentially Methylated Regions of LS-CRCs, MMR-proficient CRCs, sporadic MLH1 methylated CRCs and double somatic MMR CRCs when compared with the primary and secondary *MLH1* epimutation CRCs. Venn diagram shows the number of hypermethylated DMRs and Venn diagram showing the number of hypomethylated DMRs in common with the *MLH1* epimutation group.

**Additional file 8**. **Figure S7**: Pedigrees for each the six people in the diagnostically challenging group comprised of the *MLH1*: c.-11C>T, *MLH1*: c.-[28A>G; 7C>T] VUS carriers, and the three people with *MLH1 *methylated EOCRCs.

**Additional file 9**. **Figure S8**: Loss of heterozygosity plots in the tumours of the *MLH1*: c.-11C>T VUS carrier and the *MLH1* methylated EOCRC across the *MLH1* locus. Points represent somatic and germline variants plotted by genomic position and variant allele fraction, where green circles/shading support LOH of the region. C showing the variants across the *MLH1* locus in tumour samples from one of the *MLH1* methylated EOCRCs without LOH.

**Additional file 10**. **Figure S9**: Droplet digital PCR results in representative samples illustrated as “1D Amplitude” plots. Methylation positive and negative droplets are shown in blue and green dots, respectively. A illustrates *MLH1* methylation results in samples demonstrating negative *MLH1 *methylation, hypermethylation of *MLH1*, and low mosaic methylation in *MLH1* methylated EOCRC and *MLH1*: c.-11C>T VUS carrier. Similarly, B shows *MLH1* methylation patterns in normal and buccal-derived DNA samples.

**Additional file 11**. **Figure S10**: Pyrosequencing profiles of tumour DNA samples underwent for SMART-PCR to assess monoallelic methylation pattern of the *MLH1* promoter. A-B, CRCs from two heterozygous *MLH1*: c.-11C>T VUS carriers showing occurrent *MLH1* promoter methylation specifically in the variant allele. C-D, two reference sporadic *MLH1* methylated CRCs without the c.-11C>T variant and hence showing sole amplification of G reference allele. E, one *MLH1* methylated EOCRC that was heterozygous for the c.-93G>A promoter SNP showing sole amplification of the A SNP allele indicating monoallelic methylation associated only with this SNP allele. F-G, two sporadic *MLH1* methylated CRCs that both were heterozygous for the c.-93G>A SNP showing the amplification of both G and A alleles indicating biallelic methylation of both A and G alleles at *MLH1*: c.-93 locus.

**Additional file 12**. **Supplementary data: **Raw consensus matrix values derived from the Consensus clustering analysis on 77,113 most variably methylated CpGs, which was performed on 44 CRCs including the 6 diagnostically challenging CRCs.

## Data Availability

Genome-wide DNA methylation data (HumanMethylationEPIC array) has been deposited to GEO and accessible through Accession No. GSE233854.

## Abbreviations

ANGELS: Applying Novel Genomics approaches to Early-onset and suspected Lynch Syndrome colorectal and endometrial cancers
ACCFR: Australasian Colorectal Cancer Family Registry
CRC: Colorectal cancer
ddPCR: Droplet digital PCR
DMR: Differentially methylated region
EOCRC: Early-onset colorectal cancer
FFPE: Formalin-fixed paraffin-embedded
HMEPIC: HumanMethylationEPIC
IHC: Immunohistochemical staining
LS: Lynch syndrome
MSI: Microsatellite instability
MMR: DNA mismatch repair
MMRd: MMR-deficient
MMRp: MMR-proficient
PCR: Polymerase chain reaction
SBS: Single base substitution
TMB: Tumour mutational burden
TMS: Tumour mutational signatures
VUS: Variants of uncertain clinical significance
WES: Whole exome sequencing
