# Supplementary figures and images for: Identifying primary and secondary MLH1 epimutation carriers displaying low-level constitutional MLH1 methylation using droplet digital PCR and genome-wide DNA methylation profiling of colorectal cancers
Source: Clin Epigenetics. 2023 Jun 3;15:95. doi: 10.1186/s13148-023-01511-y (PMC10239107; doi:10.1186/s13148-023-01511-y)

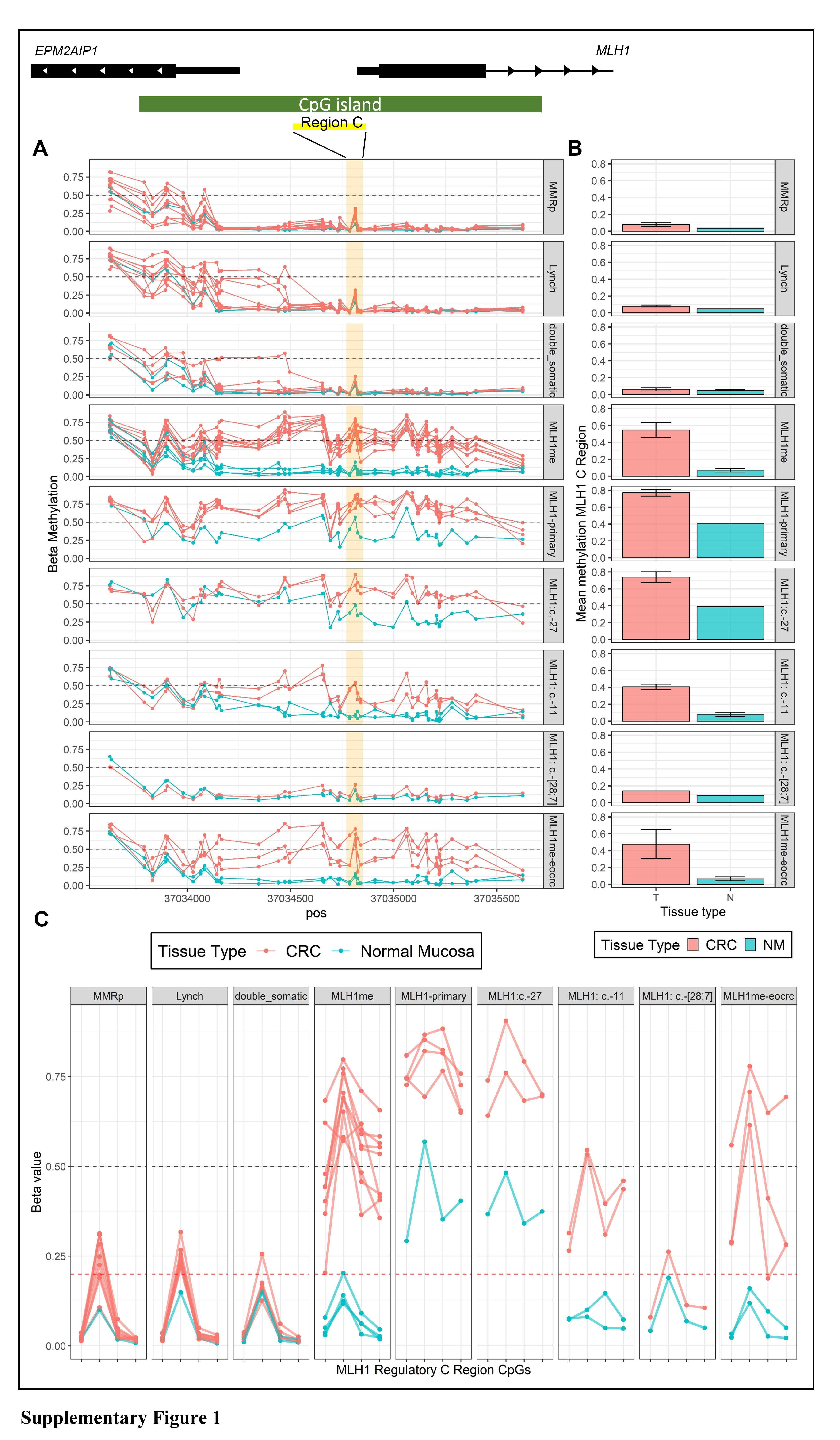

Supplement: Supplementary file 2 — Additional file 2. Figure S1:DNA methylation levels across the CpG island associated with the MLH1 promoter by CRC subgroups: 1) MMR-proficient CRCs, 2) Lynch-syndrome associated CRCs, 3) double MMR somatic mutation CRCs, 4) sporadic MLH1 methylated CRCs, 5) constitutional primary MLH1 epimutation CRCs and 6) constitutional secondary MLH1 epimutation CRCs, as well as two diagnostically challenging CRC groups: 1) MLH1 promoter VUS carriers with MLH1: c.-11C>T and MLH1: c.-[28A>G; 7C>T] and 2) MLH1 methylated EOCRCs. DNA methylation of tumour-derived DNA are shown in red and normal mucosal DNA is shown in blue. All normal mucosa samples were from resection margin, not adjacent to the tumour. Yellow boxes denote the regulatory C region as described in Deng et al. Bar plots showing mean methylation within the regulatory C region of MLH1 gene promoter across all samples for each CRC group. Tumour and normal mucosal samples are separately shown. Error bars denote standard deviation. Close-up view of four CpGs overlapping the regulatory C region. [file 13148_2023_1511_MOESM2_ESM.png]

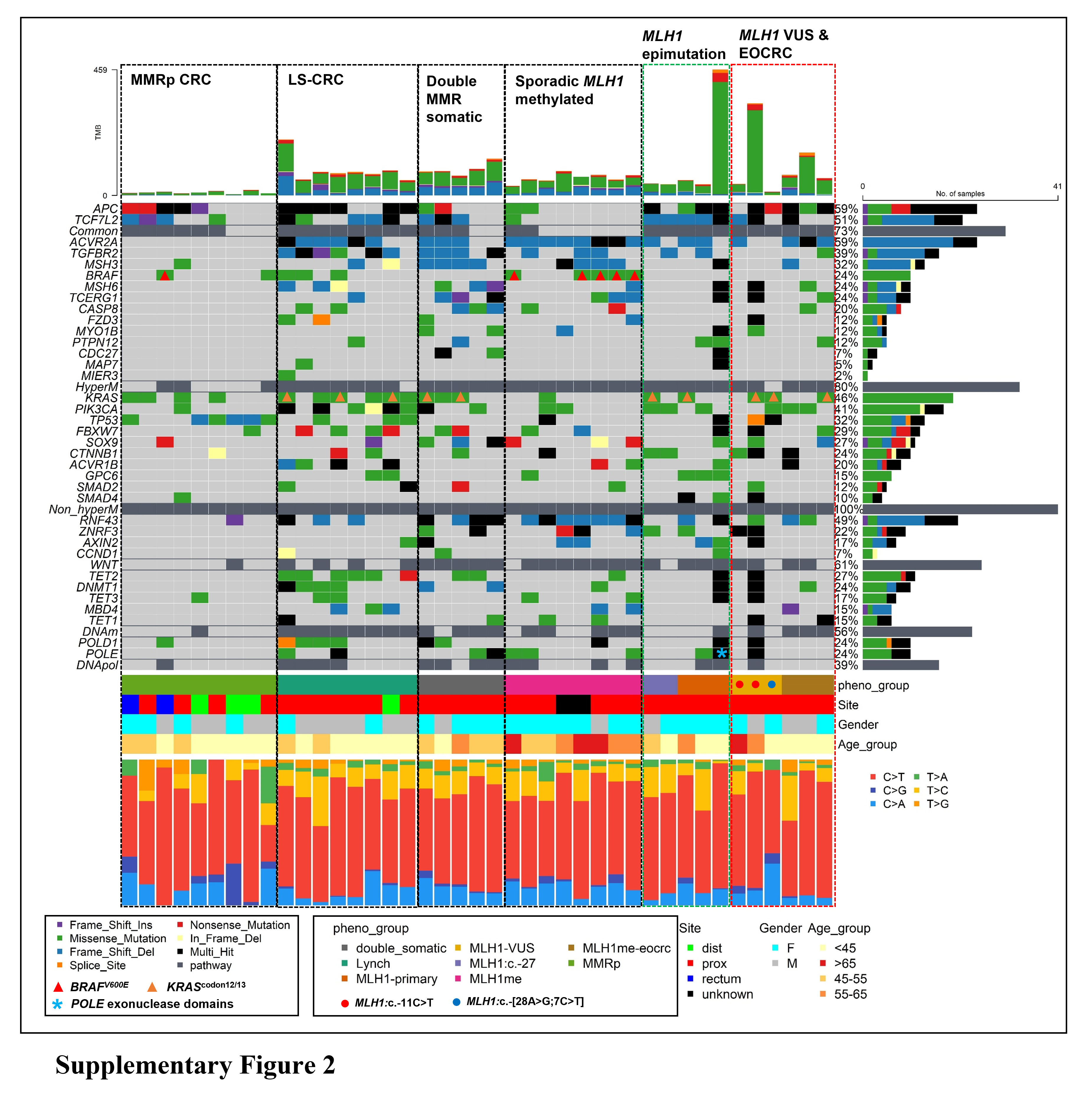

Supplement: Supplementary file 3 — Additional file 3. Figure S2: Oncoplot showing somatic mutational profiles at the 26 recurrently mutated CRC genes identified in CRC tumours from TCGA, 4 additional Wnt pathway associated genes and 5 genes related to the DNA methylation machinery. The total mutational burden shows the accumulative numbers of somatic mutations identified in each tumour sample. Individual tumour samples are further annotated by CRC subgroup, anatomical location, gender and CRC diagnosis age. The different types of somatic mutations are shown in different colours and the compositional barplots illustrate the total loads of somatic mutations separated by the nucleotide changes. One primary MLH1 epimutation CRC had a POLE somatic mutation in the exonuclease domain and also had the highest TMB with 459 total somatic mutations. [file 13148_2023_1511_MOESM3_ESM.png]

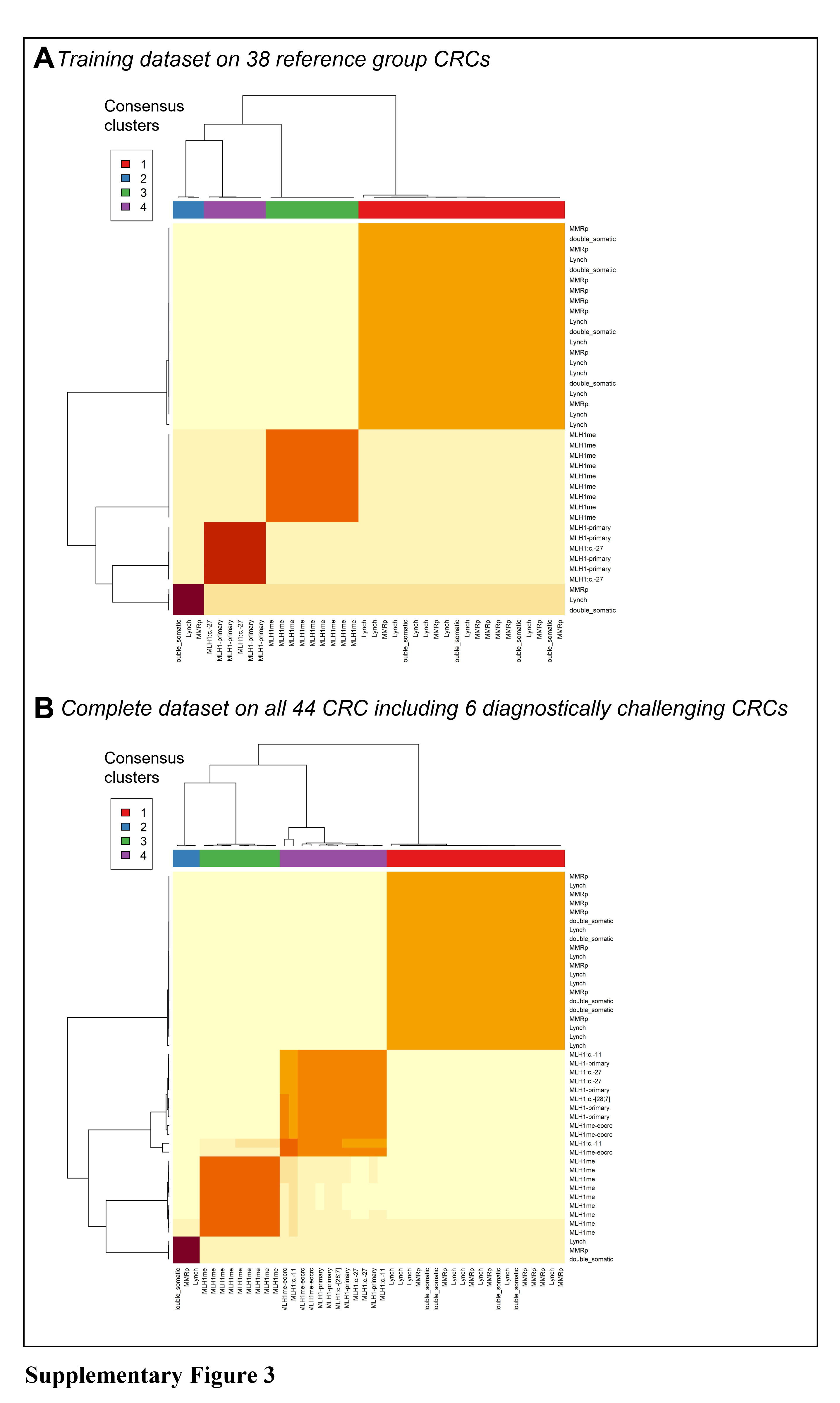

Supplement: Supplementary file 4 — Additional file 4. Figure S3: Heatmaps illustrating the consensus matrix identified by the ConsensuClusterPlus analysis. A illustrates the consensus matrix estimated on 38 reference group CRCs. B illustrates the same analysis but performed on the complete dataset of 44 CRCs including six diagnostically challenging CRCs. The final consensus cluster classification for individual samples are shown in four different colours. The darker heatmaps indicate the stability evidence for classifying individual samples into each cluster. The raw consensus values are provided in Supplementary data. [file 13148_2023_1511_MOESM4_ESM.png]

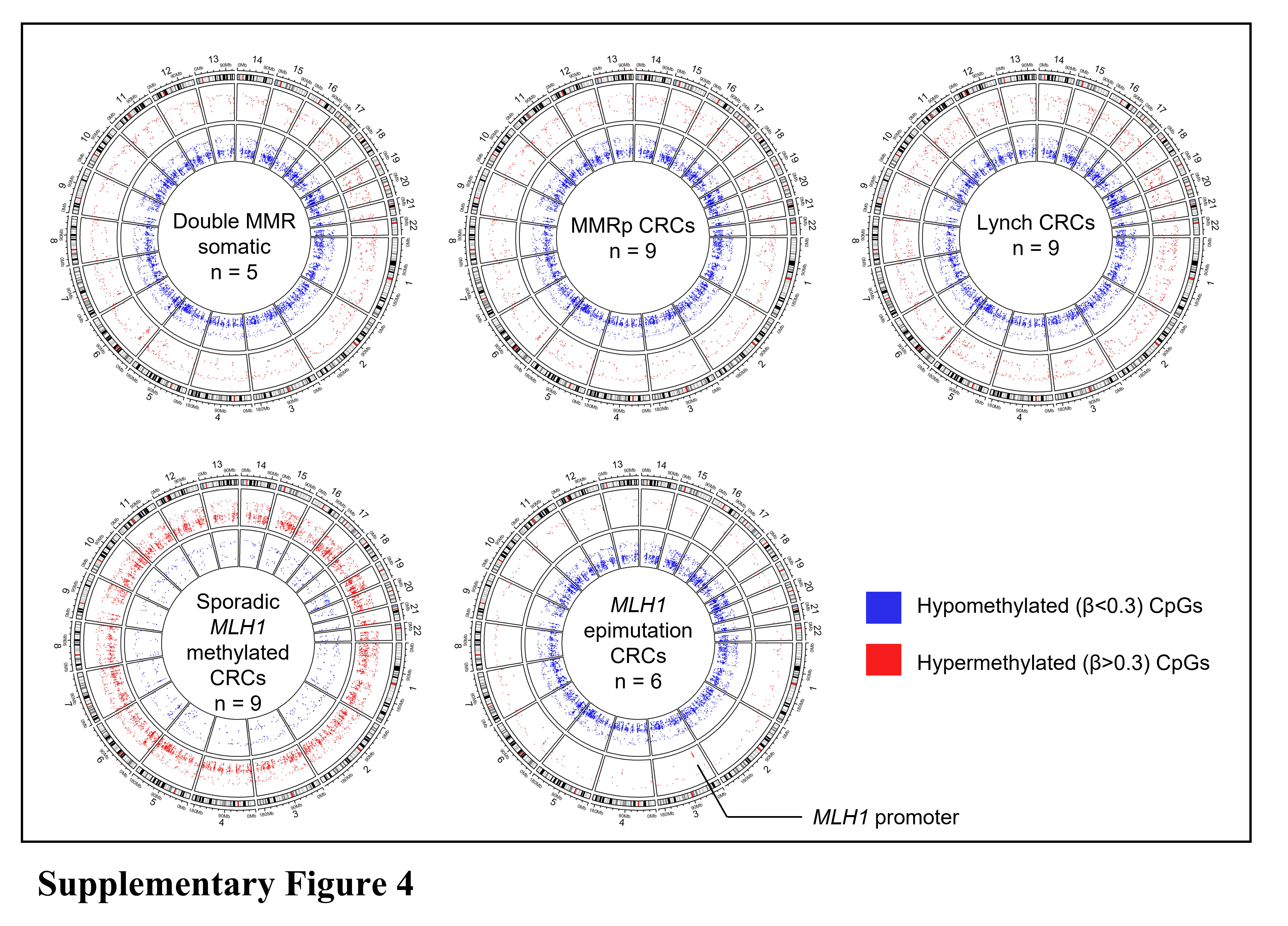

Supplement: Supplementary file 5 — Additional file 5. Figure S4: “Circos” plots showing mean genomic hypomethylation and hypermethylation patterns in tumours of five reference CRC groups. For the MLH1 epimutation group, five primary and two secondary epimutation CRCs were combined in this analysis. Mean methylation values are shown per each CRC group. [file 13148_2023_1511_MOESM5_ESM.png]

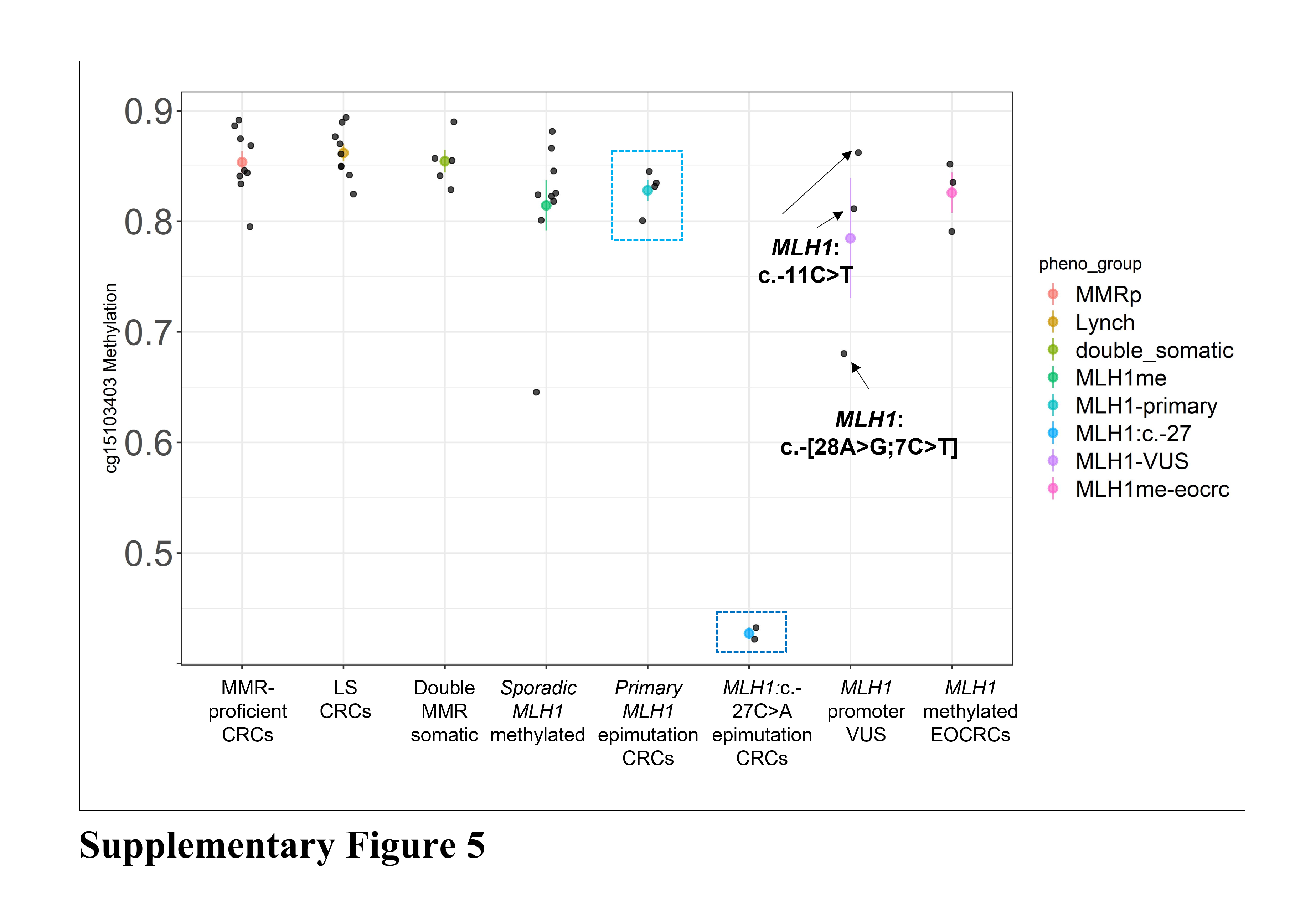

Supplement: Supplementary file 6 — Additional file 6. Figure S5: DNA methylation patterns at CpG site cg15103403 within LRRFIP2 gene for each of the six reference CRC subgroups and the two diagnostically challenging CRC subgroups. [file 13148_2023_1511_MOESM6_ESM.png]

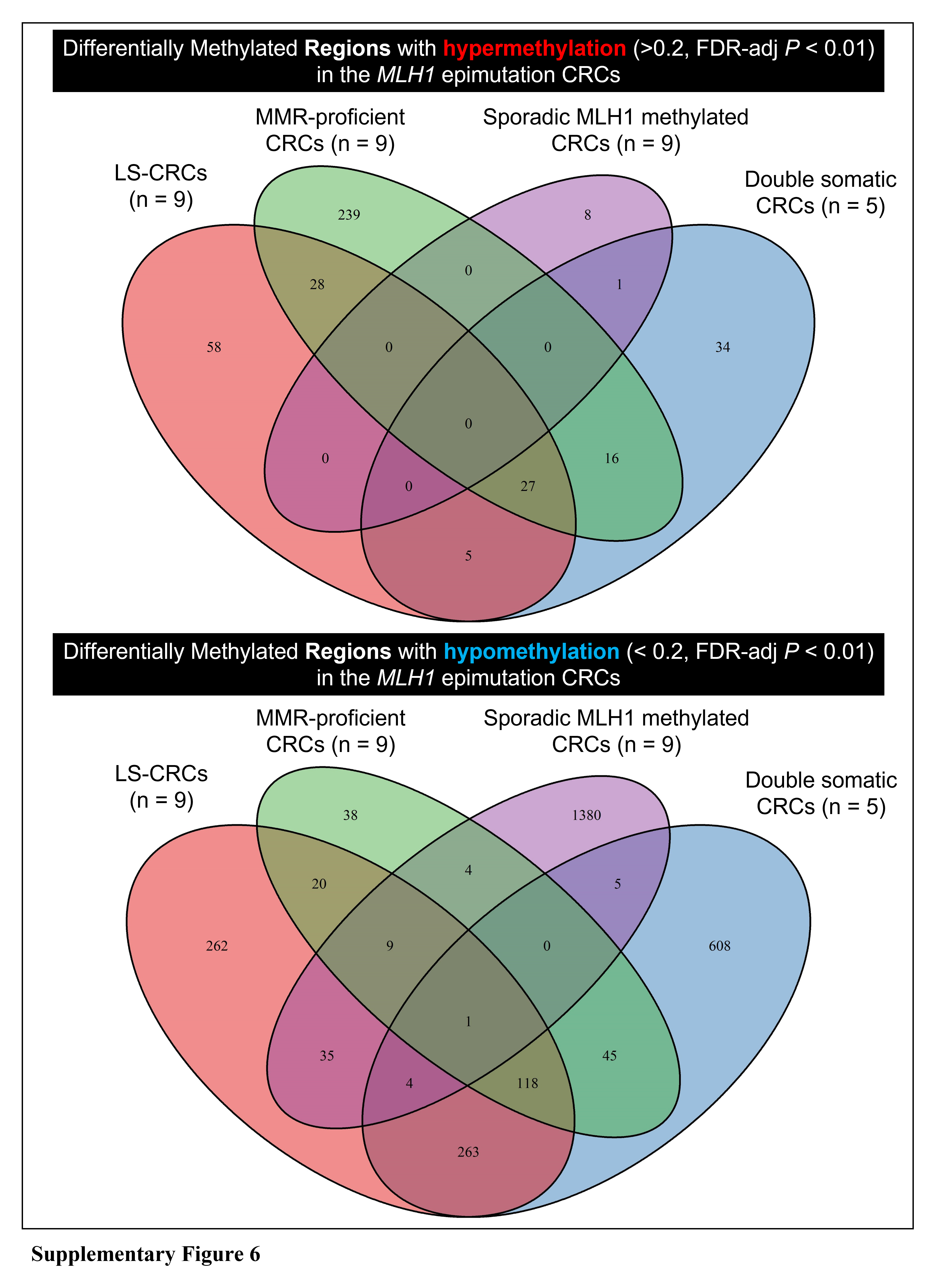

Supplement: Supplementary file 7 — Additional file 7. Figure S6: Venn diagrams showing numbers of Differentially Methylated Regions of LS-CRCs, MMR-proficient CRCs, sporadic MLH1 methylated CRCs and double somatic MMR CRCs when compared with the primary and secondary MLH1 epimutation CRCs. Venn diagram shows the number of hypermethylated DMRs and Venn diagram showing the number of hypomethylated DMRs in common with the MLH1 epimutation group. [file 13148_2023_1511_MOESM7_ESM.png]

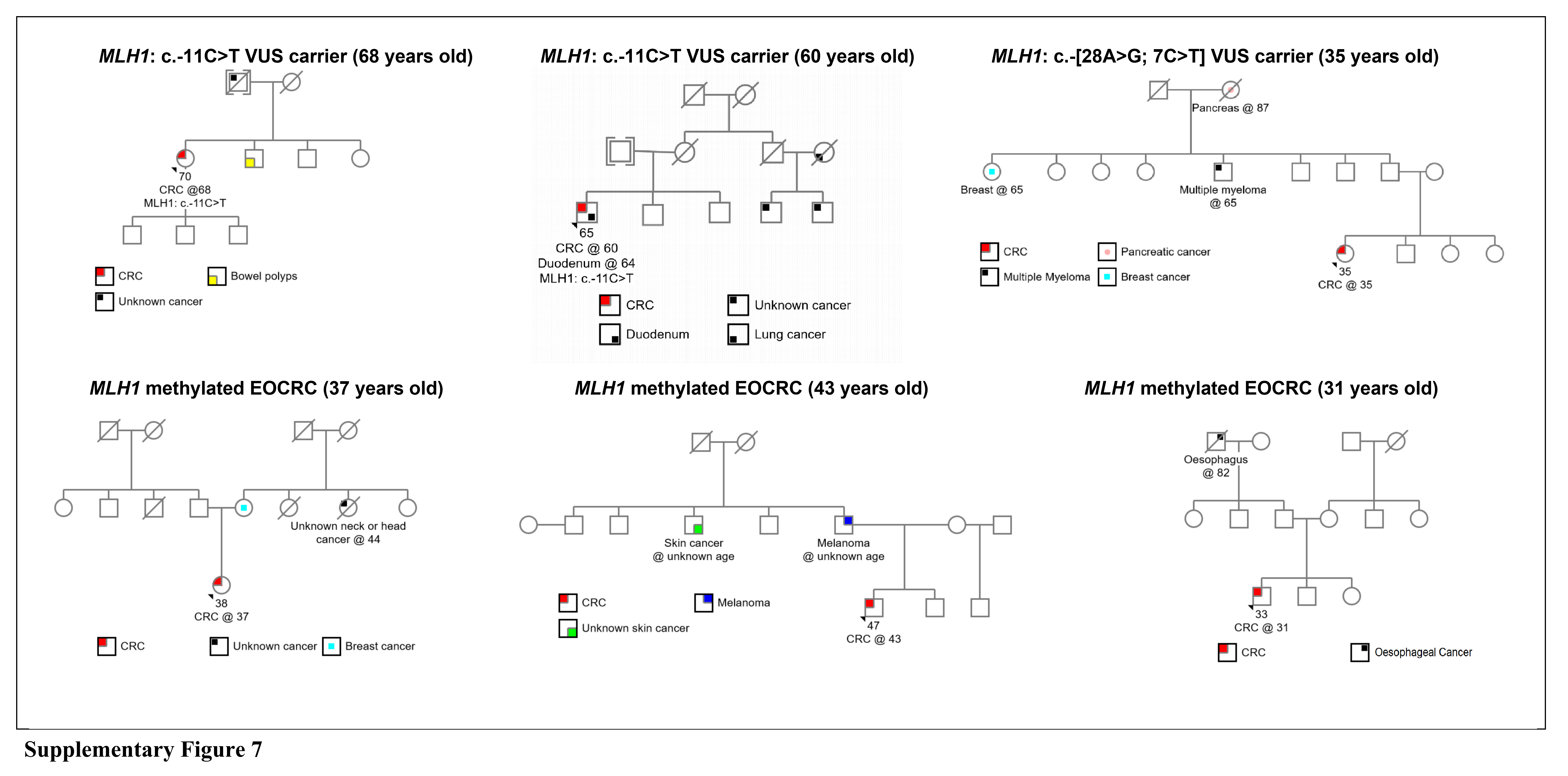

Supplement: Supplementary file 8 — Additional file 8. Figure S7: Pedigrees for each the six people in the diagnostically challenging group comprised of the MLH1: c.-11C>T, MLH1: c.-[28A>G; 7C>T] VUS carriers, and the three people with MLH1 methylated EOCRCs. [file 13148_2023_1511_MOESM8_ESM.png]

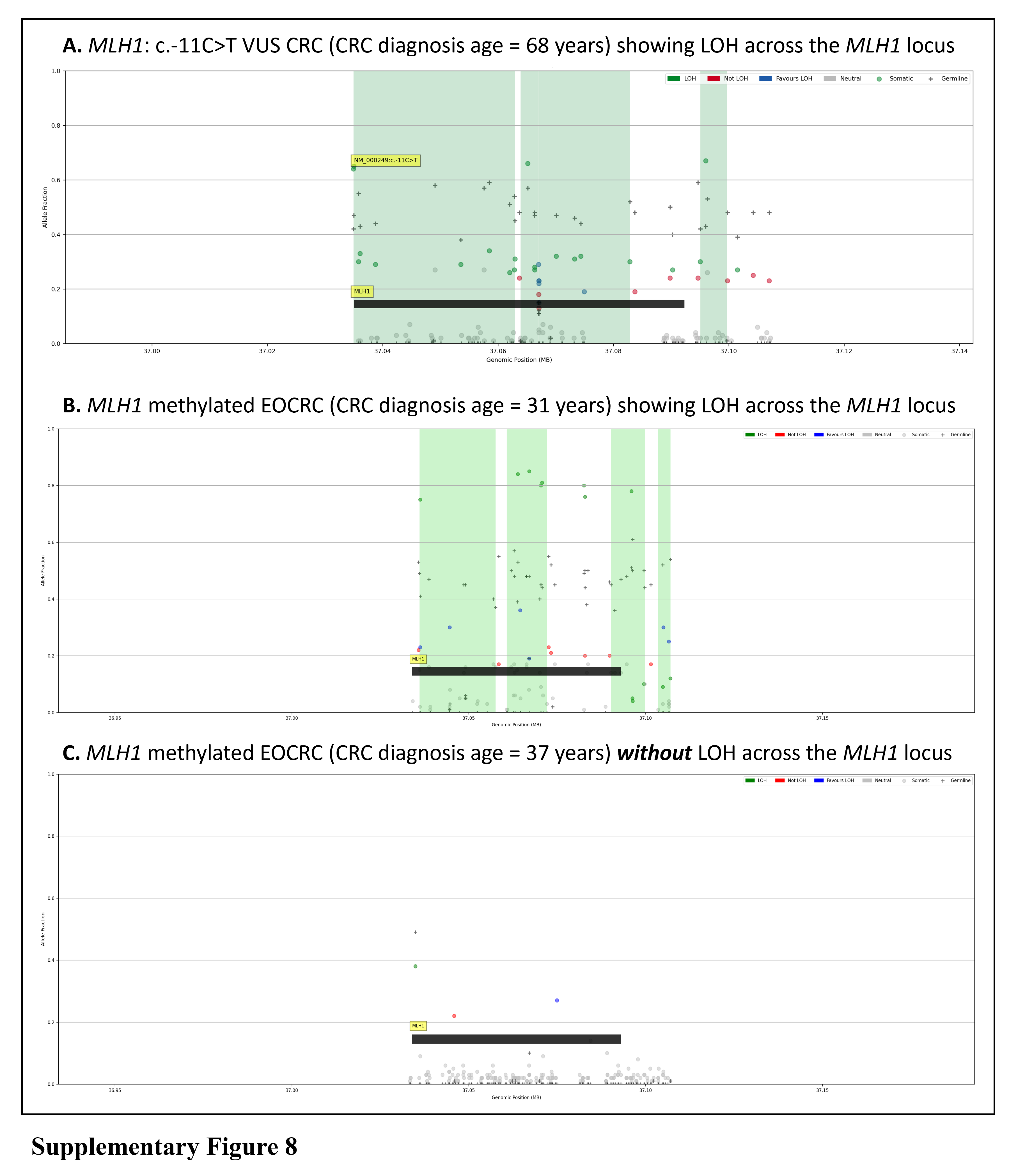

Supplement: Supplementary file 9 — Additional file 9. Figure S8: Loss of heterozygosity plots in the tumours of the MLH1: c.-11C>T VUS carrier and the MLH1 methylated EOCRC across the MLH1 locus. Points represent somatic and germline variants plotted by genomic position and variant allele fraction, where green circles/shading support LOH of the region. C showing the variants across the MLH1 locus in tumour samples from one of the MLH1 methylated EOCRCs without LOH. [file 13148_2023_1511_MOESM9_ESM.png]

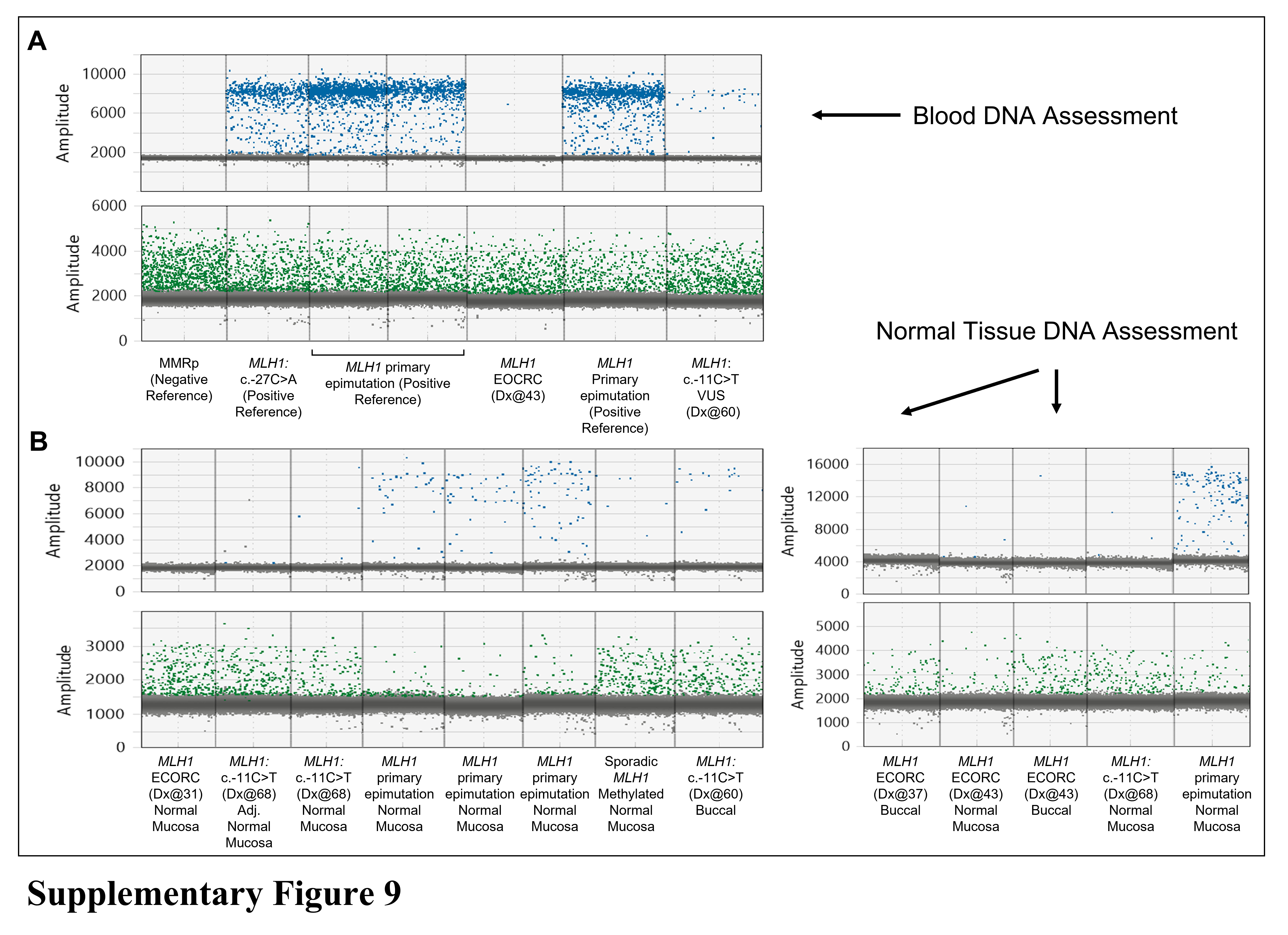

Supplement: Supplementary file 10 — Additional file 10. Figure S9: Droplet digital PCR results in representative samples illustrated as “1D Amplitude” plots. Methylation positive and negative droplets are shown in blue and green dots, respectively. A illustrates MLH1 methylation results in samples demonstrating negative MLH1 methylation, hypermethylation of MLH1, and low mosaic methylation in MLH1 methylated EOCRC and MLH1: c.-11C>T VUS carrier. Similarly, B shows MLH1 methylation patterns in normal and buccal-derived DNA samples. [file 13148_2023_1511_MOESM10_ESM.png]

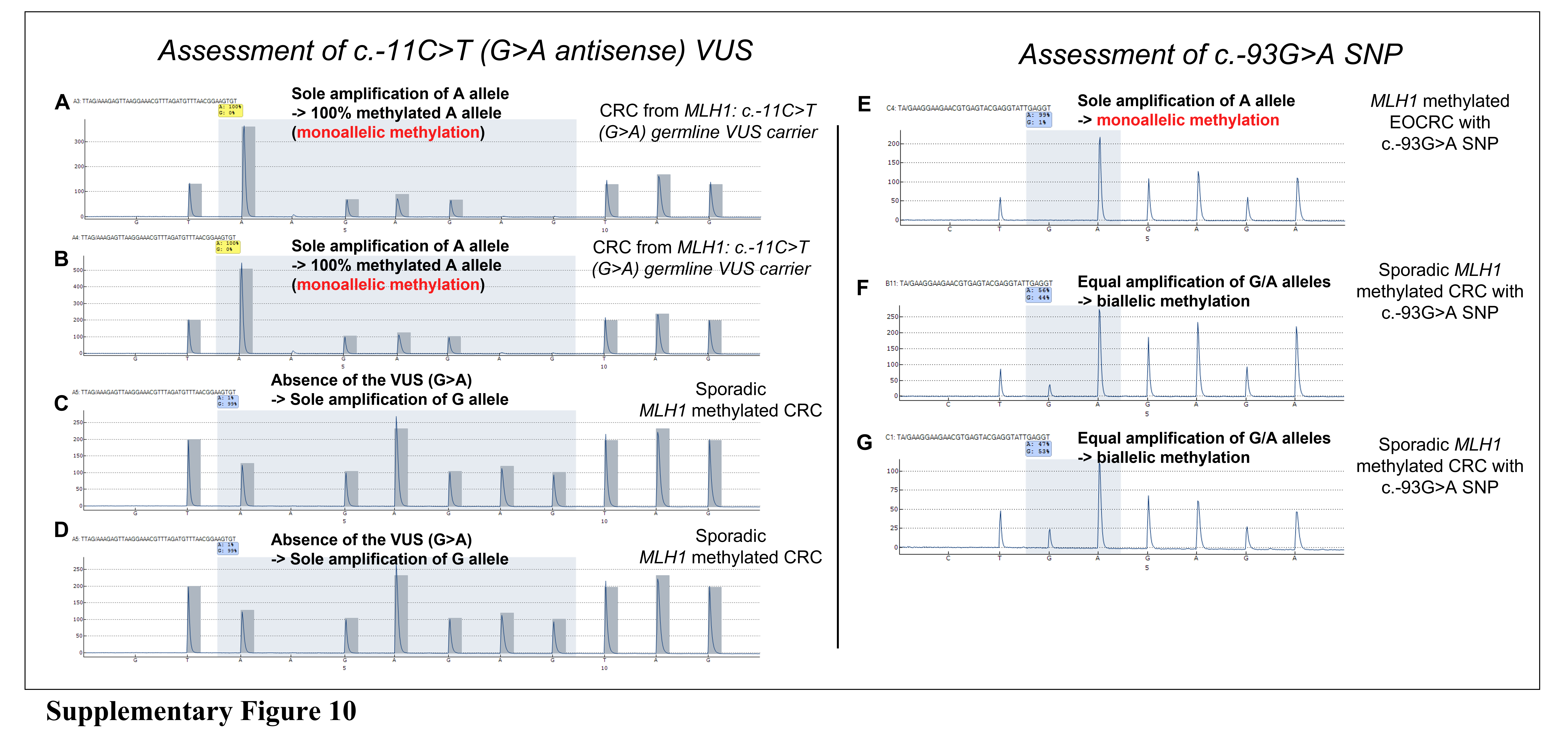

Supplement: Supplementary file 11 — Additional file 11. Figure S10: Pyrosequencing profiles of tumour DNA samples underwent for SMART-PCR to assess monoallelic methylation pattern of the MLH1 promoter. A-B, CRCs from two heterozygous MLH1: c.-11C>T VUS carriers showing occurrent MLH1 promoter methylation specifically in the variant allele. C-D, two reference sporadic MLH1 methylated CRCs without the c.-11C>T variant and hence showing sole amplification of G reference allele. E, one MLH1 methylated EOCRC that was heterozygous for the c.-93G>A promoter SNP showing sole amplification of the A SNP allele indicating monoallelic methylation associated only with this SNP allele. F-G, two sporadic MLH1 methylated CRCs that both were heterozygous for the c.-93G>A SNP showing the amplification of both G and A alleles indicating biallelic methylation of both A and G alleles at MLH1: c.-93 locus. [file 13148_2023_1511_MOESM11_ESM.png]
